# Potential of acetic acid to restore methane production in anaerobic reactors critically intoxicated by ammonia as evidenced by metabolic and microbial monitoring

**DOI:** 10.1186/s13068-023-02438-5

**Published:** 2023-12-02

**Authors:** Sébastien Lemaigre, Patrick A. Gerin, Gilles Adam, Dominika Klimek, Xavier Goux, Malte Herold, Zuzana Frkova, Magdalena Calusinska, Philippe Delfosse

**Affiliations:** 1 Environmental Research and Innovation Department, Luxembourg Institute of Science and Technology, Rue du Brill 41, L-4422 Belvaux, Luxembourg; 2 Earth and Life Institute, Bioengineering, Université Catholique de Louvain, Croix du Sud 2, Box L7.05.19, B-1348 Louvain-la-Neuve, Belgium; 3 Université du Luxembourg, Campus Belval, Maison du Savoir, Avenue de l’Université 2, L-4365 Esch-sur-Alzette, Luxembourg

**Keywords:** Anaerobic digestion, Free ammonia nitrogen intoxication, Process recovery, Microbial community monitoring, Restoration strategy

## Abstract

**Background:**

Biogas and biomethane production from the on-farm anaerobic digestion (AD) of animal manure and agri-food wastes could play a key role in transforming Europe’s energy system by mitigating its dependence on fossil fuels and tackling the climate crisis. Although ammonia is essential for microbial growth, it inhibits the AD process if present in high concentrations, especially under its free form, thus leading to economic losses. In this study, which includes both metabolic and microbial monitoring, we tested a strategy to restore substrate conversion to methane in AD reactors facing critical free ammonia intoxication.

**Results:**

The AD process of three mesophilic semi-continuous 100L reactors critically intoxicated by free ammonia (> 3.5 g_N L^−1^; inhibited hydrolysis and heterotrophic acetogenesis; interrupted methanogenesis) was restored by applying a strategy that included reducing pH using acetic acid, washing out total ammonia with water, re-inoculation with active microbial flora and progressively re-introducing sugar beet pulp as a feed substrate. After 5 weeks, two reactors restarted to hydrolyse the pulp and produced CH_4_ from the methylotrophic methanogenesis pathway. The acetoclastic pathway remained inhibited due to the transient dominance of a strictly methylotrophic methanogen (*Candidatus* Methanoplasma genus) to the detriment of *Methanosarcina*. Concomitantly, the third reactor, in which *Methanosarcina* remained dominant, produced CH_4_ from the acetoclastic pathway but faced hydrolysis inhibition. After 11 weeks, the hydrolysis, the acetoclastic pathway and possibly the hydrogenotrophic pathway were functional in all reactors. The methylotrophic pathway was no longer favoured. Although syntrophic propionate oxidation remained suboptimal, the final pulp to CH_4_ conversion ratio (0.41 ± 0.10 L_N__CH_4_ g_VS^−1^) was analogous to the pulp biochemical methane potential (0.38 ± 0.03 L_N__CH_4_ g_VS^−1^).

**Conclusions:**

Despite an extreme free ammonia intoxication, the proposed process recovery strategy allowed CH_4_ production to be restored in three intoxicated reactors within 8 weeks, a period during which re-inoculation appeared to be crucial to sustain the process. Introducing acetic acid allowed substantial CH_4_ production during the recovery period. Furthermore, the initial pH reduction promoted ammonium capture in the slurry, which could allow the field application of the effluents produced by full-scale digesters recovering from ammonia intoxication.

**Supplementary Information:**

The online version contains supplementary material available at 10.1186/s13068-023-02438-5.

## Background

The European Union (EU) is currently proposing to increase the 2030 target for renewable energy source consumption to 45% [1]. In this context, biogas production from the on-farm anaerobic digestion (AD) of animal manure and agri-food residues can play a key role in increasing the contribution of alternative energy sources. AD can help accelerate the transition towards net-zero global greenhouse gas emissions by 2050 while valorizing carbon and nitrogen from biodegradable waste resources [2]. Moreover, biogas production increases European energy security by reducing dependence on imported fossil fuels and can alleviate the burden of energy costs on households and industries [3].

Biogas production through AD has increased almost fivefold in the last two decades in the EU [4], especially in Germany, where national subsidies promoted biogas production relying on the use of energy crops as the main reactor feedstocks [5]. However, competition for land between energy and food markets resulted in food versus fuel debates [6]. A comparative assessment, employing parameters, such as fossil fuel consumption, greenhouse gas emissions, fertilizer application, electricity consumption and transportation—as provided by the EU document on the sustainability of solid and gaseous biomass used for bioenergy production [7] revealed that biogas production from agri-food wastes could generate ~ 30% less CO_2_ emissions than biogas production from energy crops [8]. Therefore, the replacement of energy crops with agri-food wastes in biogas production through AD is both environmentally sustainable and economically profitable in the long term. However, changing the feedstock in AD to nitrogen-rich substrates (manure, organic fraction of municipal solid wastes or waste from abattoirs) could result in both acute and chronic ammonia toxicities for the reactors’ microbial communities [9, 10].

Total ammonia nitrogen (TAN) exists in two forms in aqueous environments, such as AD slurries: ionized ammonium nitrogen (NH_4_^+^–N) and un-ionized “free” ammonia nitrogen (FAN, i.e., NH_3_–N). The concentration ratio of these two forms is driven by pH and temperature [11]. FAN is generally regarded as the main cause of the inhibition of the methanogenesis [12] of nitrogen-rich substrates due to its high diffusion rate through membranes of microbial cells [11]. Methanogenic archaea are especially vulnerable to ammonia intoxication compared to the other AD microbes because of their cell wall composition, which lacks peptidoglycan [13, 14]. At an equivalent pH, the FAN proportion is higher in thermophilic temperature conditions that in mesophilic conditions. Therefore, thermophilic archaea were reported to have developed a higher tolerance to FAN than the mesophilic ones [15]. Indeed, the former are more likely to live at higher FAN concentrations than the latter. In uninhibited AD reactors, it is generally assumed that ~ 70% of methanogenesis is attributed to the acetoclastic pathway and ~ 30% to the hydrogenotrophic pathway [16, 17], while only a minimal amount of CH_4_ (< 1%) is produced from methyl compounds via methylotrophic methanogenesis [18]. Under a high FAN concentration in the slurry, hydrogenotrophic methanogens were reported to become predominant and accordingly, the major methanogenesis pathway shifted to hydrogenotrophic [19, 20]. The same authors also reported the presence of bacterial syntrophic acetate oxidizers (SAO) in association with these hydrogenotrophic archaea. In FAN-intoxicated reactors relying mostly on the hydrogenotrophic pathway, any disturbance to methanogenesis would cause an accumulation of hydrogen in the slurry, resulting in a thermodynamic blockage of propionate degradation over a timescale of 1 s [21]. This phenomenon could explain why AD reactors treating nitrogen-rich substrate are subject to a quick and often irreversible process failure [11]. Therefore, excessive FAN content in the slurry strongly affects the performance of AD reactors, causing significant economic losses for biogas plants. The ideal way to address these economic losses is to ensure a careful substrate management including balanced nitrogen availability within the reactors. However, this is difficult to put into practice on farms, as the substrate composition varies on a daily/weekly basis, and quantities are fed arbitrarily. Therefore, it is necessary to detect the risk of process dysfunction and put prevention systems in place [22]. However, preventive measures are challenging to implement as the FAN toxicity depends on the complexity of the AD process, where mechanisms such as antagonism, synergism and acclimatization can significantly affect the phenomenon of inhibition [11, 23, 24].

Various management methods have been investigated to avoid or mitigate FAN intoxication in AD reactors, including pH alteration with hydrochloric or humic acid, the adjustment of the feedstock C/N ratio, slurry dilution and microbial immobilization [9, 25, 26]. Besides these potential solutions, a few strategies have also recently been investigated, including the gradual acclimation of the microorganisms to high nitrogen conditions [23] and lignite addition for ammonium adsorption [12]. However, these methods aim either at preventing ammonia inhibition or at optimizing biogas production after a moderate ammonia intoxication, and studies proposing recovery strategies after critical FAN-intoxication of the AD process are scarce [27, 28].

**Fig. 1:**
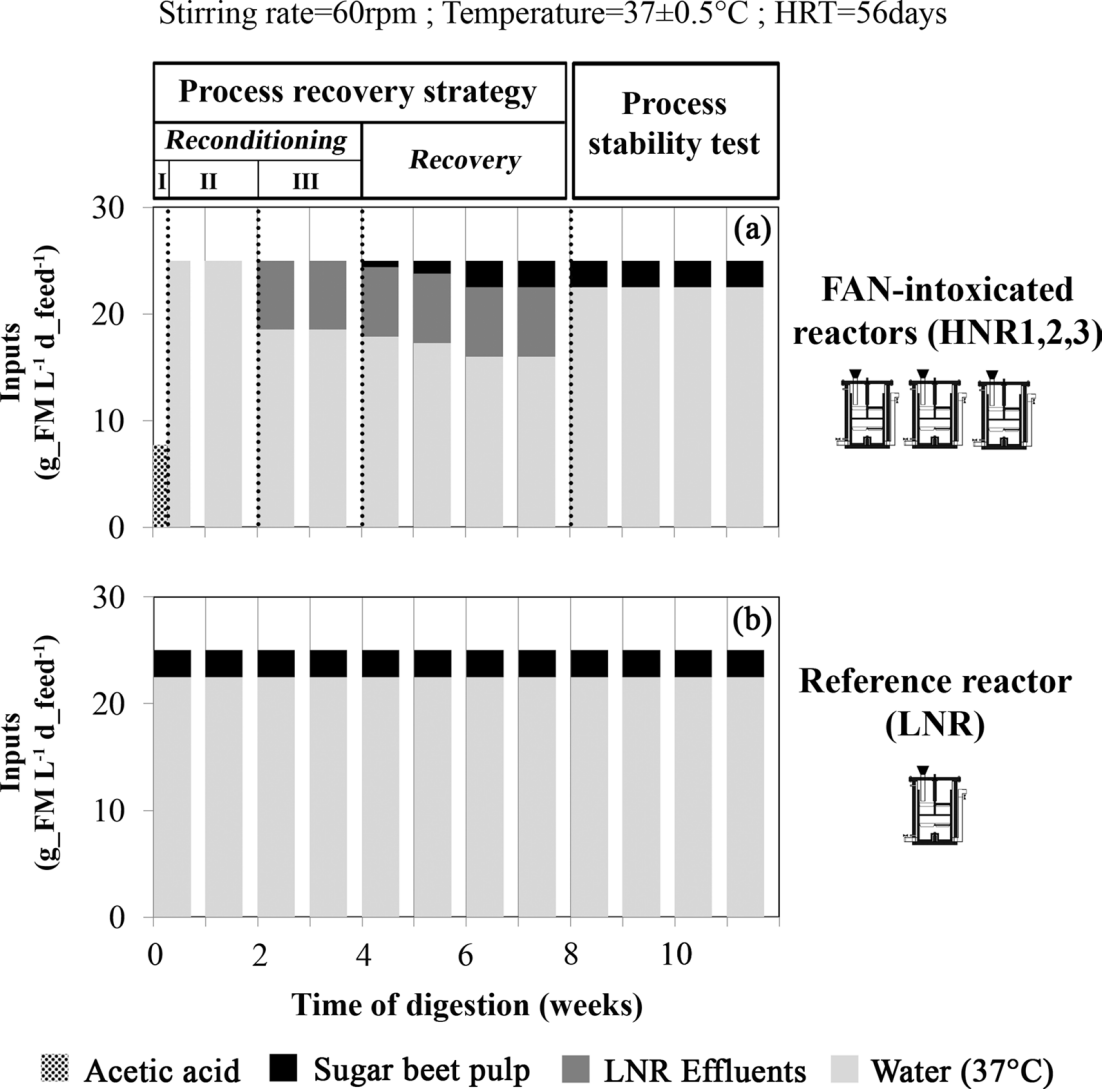
Mass of inputs (expressed in fresh matter, FM) introduced per litre of slurry and per feeding day (i.e., d_feed) in (**a**) the free ammonia intoxicated reactors, HNRs and (**b**) the reference reactor, LNR. “Process reconditioning I, II, III”, “Process recovery” and “Process stability test” refer to the stages and phases described in Table 1

This paper complements our previous work on the assessment of a multivariate statistical process control model exploiting biogas composition to predict the process status of AD reactors that were intentionally driven to critical FAN intoxication [24]. In the previous study, triplicate mesophilic pilot-scale continuous stirred tank reactors (referred to as the high nitrogen input reactors HNR1, HNR2 and HNR3) were fed with a sugar beet pulp basal diet complemented with increasing amounts of urea until the complete interruption of their biogas production due to FAN toxicity [FAN > 3.5 g of nitrogen per litre of slurry (i.e., g_N L^−1^); TAN > 17.5 g_N L^−1^]. In parallel, a fourth reactor (referred to as the low nitrogen input reactor, LNR), was fed with the sugar beet pulp basal diet only (no urea supplied) and was used as a reference reactor. We showed that the first warning signal delivered by our model was synchronous to a shift in the microbiome structure of the three HNRs, which announced the upcoming process collapse. In the present study, we further tested a specifically designed process recovery strategy (Table 1 and Fig. 1), which was applied to these three critically FAN-intoxicated reactors (here still referred to as HNRs). The aim was to assess whether this process recovery strategy could restore the main AD process pathways in our HNRs, in which prolonged and excessive FAN exposure made the hydrolysis, heterotrophic acetogenesis and methanogenesis unfunctional. The process status of the HNRs was assessed by monitoring key abiotic process stability indicators and comparing them to those measured for the LNR as a reference for optimal process performance. A particular focus was placed on tracing the microbial communities due to their ability to convert the introduced substrates (sugar beet pulp and acetic acid) to CH_4_. Therefore, bacteria and archaea were monitored (separately) using 16S rRNA gene amplicon high-throughput sequencing, allowing us to link changes in the ecological richness, evenness and diversity of the microbial communities to the recovery strategy applied and the final process stability assessment.

**Table 1:** Treatments applied to the free ammonia intoxicated reactors, HNR1, HNR2 and HNR3

Stage	Phase	Weeks	Treatment
Process reconditioning	I	0 (day 0–1)	*pH reduction with acetic acid* (15 g_VS L^−1^ in total)
	II	0 (days 2–5) and 1	*Dilution with tap water at 37°C* (25 mL L^−1^ d_feed^−1^)
	III	2 to 3	*Re-inoculation with LNR effluents* (6.5 mL L^−1^ d_feed^−1^); Water dilution*
Process recovery	/	4 to 7	*Re-inoculation with LNR effluents* (6.5 mL L^−1^ d_feed^−1^); *Feeding with sugar beet pulp* (0.5 → 2 g_VS L^−1^ d_feed^−1^); Water dilution*
Process stability test	/	8 to 11	*Feeding with sugar beet pulp* (2 g_VS L^−1^ d_feed^−1^); Water dilution*

The reactors were fed on working days, usually five consecutive days (i.e., d_feed) per week; * inputs suspended in tap water at 37°C to reach a hydraulic retention time of 56 days (total fed volume: 25 mL L−1 d_feed−1); LNR: low nitrogen input reactor used as a reference reactor; VS: volatile solids

## Results and discussion

### Study design and the process recovery strategy

The reactors were fed on working days, usually five consecutive days (i.e., d_feed) per week, following a semi-continuous scheme. Our process recovery started at day 0 with a 3-phase reconditioning stage during which the intoxicated HNRs were sequentially exposed to (i) pH reduction using pure acetic acid to induce a quick drop in the FAN content (reconditioning stage—phase I, first 2 days of week 0), (ii) a water dilution period (reconditioning stage—phase II, end of week 0 and week 1) and (iii) a period of re-inoculation by LNR effluents combined with moderate water dilution (reconditioning stage—phase III, weeks 2–3). After the reconditioning stage, the HNRs were fed with sugar beet pulp to progressively increase their organic loading rate (OLR) and re-inoculation by LNR effluents was maintained (process recovery stage, weeks 4–7). Finally, re-inoculation was stopped for the three HNRs but the sugar beet pulp feeding was maintained (process stability test stage, weeks 8–11).

### Process stability and microbiome of the reference reactor

**Fig. 2:**
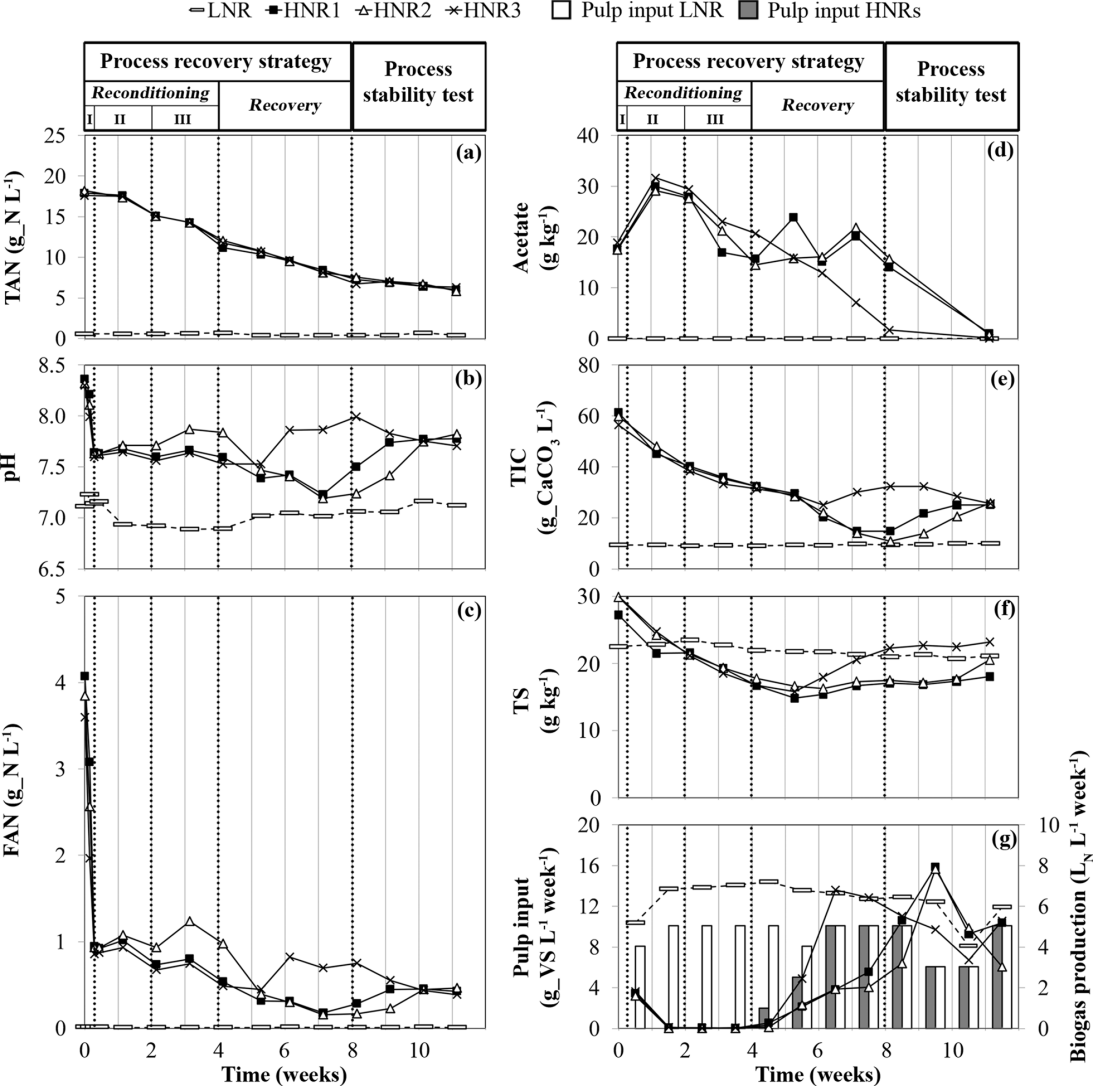
Progress over time of: **a** total ammonia nitrogen (TAN) measured; **b** pH; **c** free ammonia nitrogen (FAN) calculated; **d** acetate; **e** total inorganic carbon (TIC); **f** total solid (TS) content in the slurry of the reactors; **g** mass of sugar beet pulp (expressed in volatile solids, VS) introduced weekly into the reactors and their weekly biogas production. The decrease in biogas production observed during week 10 for the LNR reference reactor is due to a reduced pulp input in weeks 9 and 10 (only three feeding days per week). “Process reconditioning I, II, III”, “Process recovery” and “Process stability test” refer to the stages and phases described in Table 1. HNR: free ammonia intoxicated reactor

**Fig. 3:**
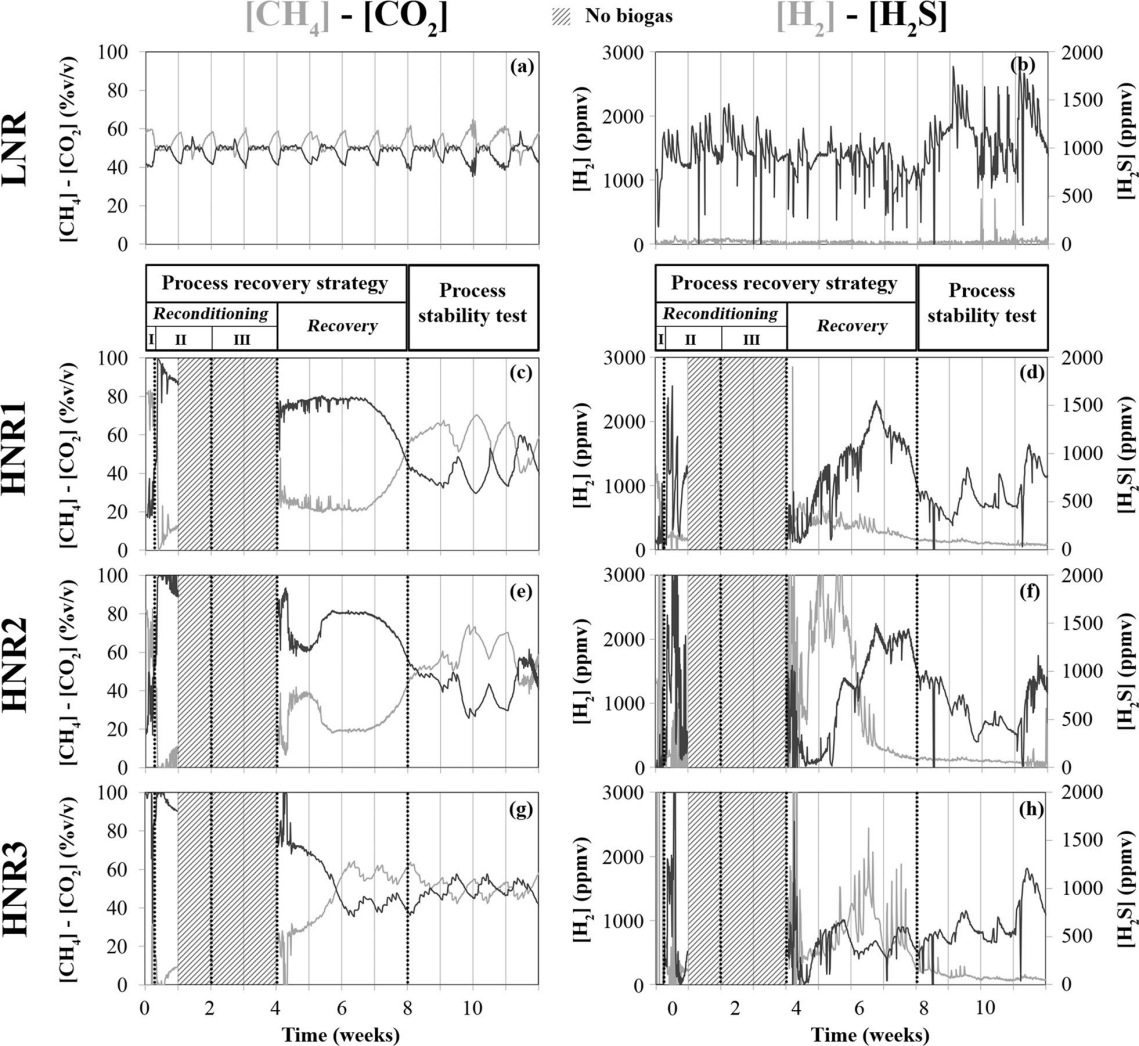
Progress over time of the biogas concentrations in CH_4_, CO_2_, H_2_ and H_2_S for: **a**, **b** the LNR reference reactor, **c**, **d** the free ammonia intoxicated reactor, HNR1, **e**, **f** HNR2 and **g**, **h** HNR3. CH_4_ and CO_2_ concentrations are expressed in volume/volume percents (%v/v). H_2_ and H_2_S concentrations are expressed in parts per million by volume (ppmv). Measurements could not be performed from week 1 to week 3 due to the interruption of the biogas production (hatched grey area). “Process reconditioning I, II, III”, “Process recovery” and “Process stability test” refer to the stages and phases described in Table 1

In the LNR reference reactor, the fed sugar beet pulp was efficiently and continuously converted to methane, with a CH_4_ yield of 0.32 ± 0.1 normalized litres (L_N_) of CH_4_ per gram of volatile solids (VS) added (i.e., L_N__CH_4_ g_VS^−1^). This value remained close to the pulp biochemical methane potential (BMP, 0.38 ± 0.03 L_N__CH_4_ g_VS^−1^), which is in accordance with the continuous but careful feeding regime of this reactor [OLR = 2 g of VS per litre of slurry and per feeding day (i.e., g_VS L^−1^ d_feed^−1^)] [24]. The biogas production was ~ 6 L_N_ L^−1^ week^−1^ (Fig. 2g) and was characterized by a [CH_4_]/[CO_2_] ratio close to 1 (Fig. 3a) and an H_2_ concentration lower than 100 parts per million by volume (ppmv) (Fig. 3b). The total volatile fatty acid (TVFA) concentration remained below 0.04 g per kilogram of slurry (i.e., g kg^−1^).

**Fig. 4:**
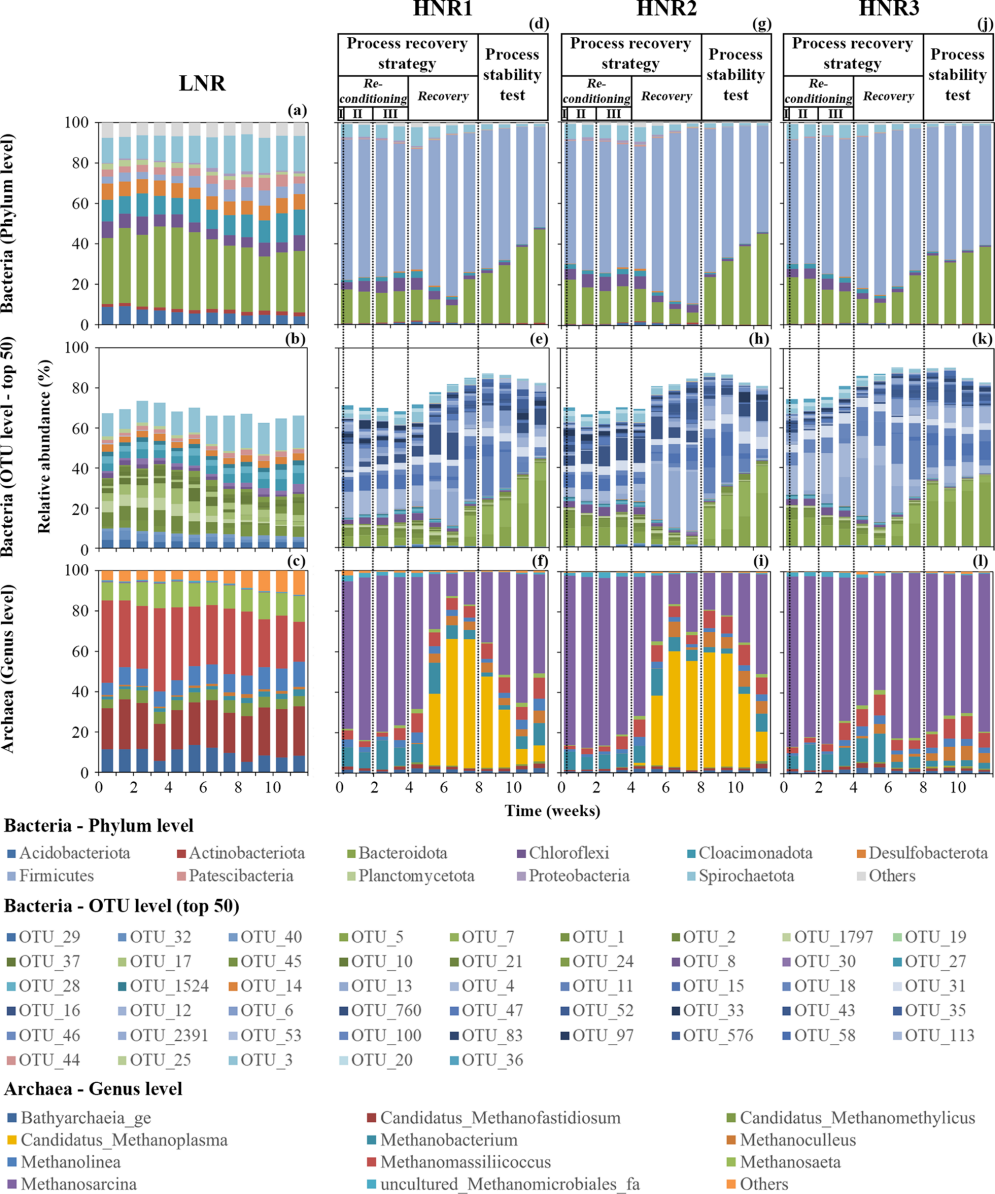
Bacterial (phylum level and top 50 OTUs level) and archaeal (genus level) structure dynamics over time as assessed by high-throughput 16S rRNA amplicon sequencing for: **a–c** the LNR reference reactor, **d**–**f** the free ammonia intoxicated reactor, HNR1, **g**–**i** HNR2 and **j**–**l** HNR3. The data presented correspond to slurry samples collected on Mondays. “Process reconditioning I, II, III”, “Process recovery” and “Process stability test” refer to the stages and phases described in Table 1. We refer to the Additional file 1: Table S1 for the taxonomic affiliation of the bacterial OTUs

**Fig. 5:**
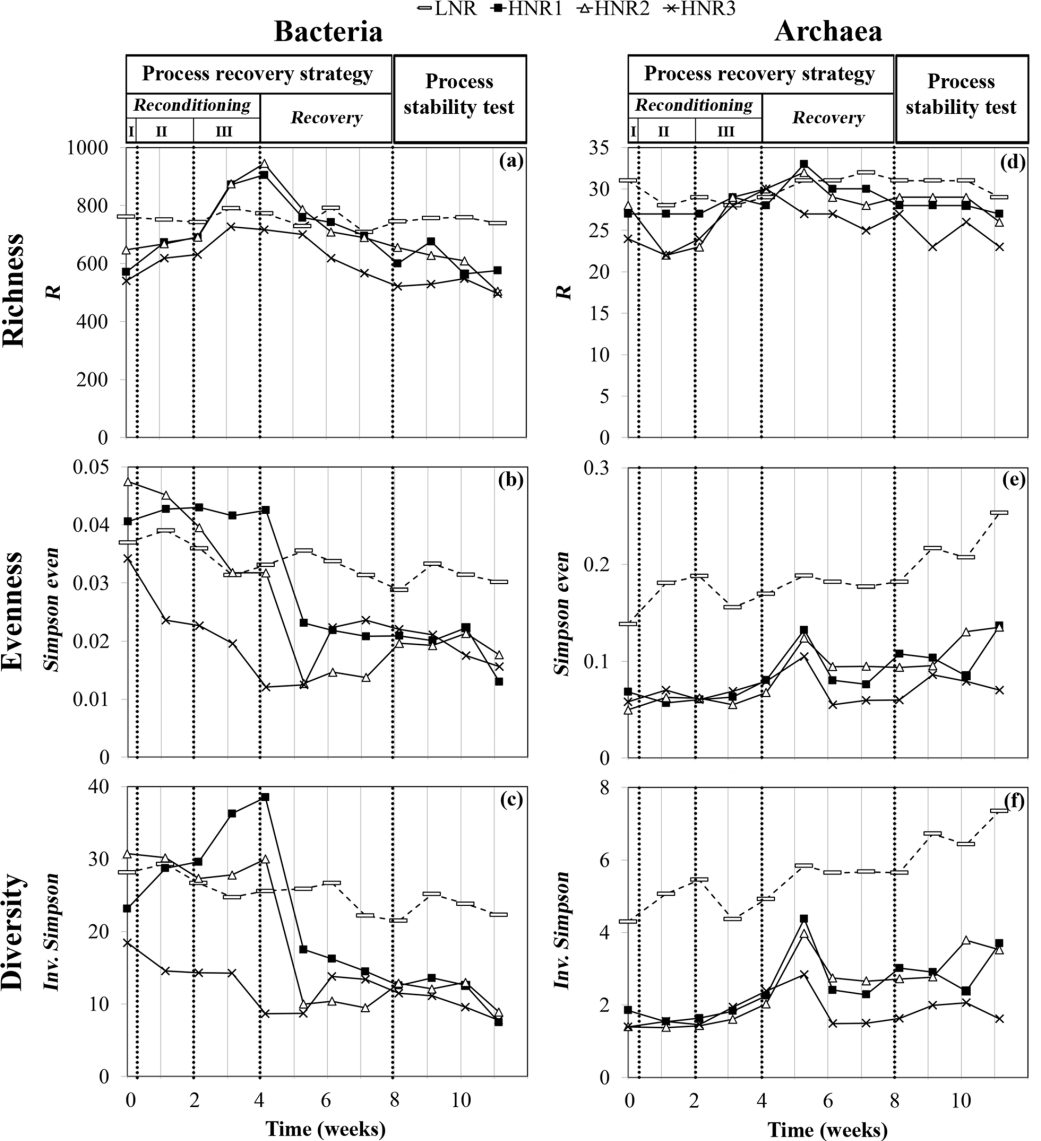
Progress over time of the ecological richness, evenness and diversity calculated at the OTU level for bacteria (**a–c**) and archaea (**d–f**) for the LNR reference reactor and the free ammonia intoxicated reactors, HNR1, HNR2 and HNR3. For the (observed) richness (*R*) and diversity (*Inv. Simpson*), increased values indicate a higher richness and diversity of species, respectively. For the evenness (*Simpson even*), values range from 0 (uneven community; one or several dominant OTUs and many singlets) to 1 (perfectly even community, all OTUs present at the same relative abundance). “Process reconditioning I, II, III”, “Process recovery” and “Process stability test” refer to the stages and phases described in Table 1

*Bacteroidota* phylum dominated the bacterial community of the LNR throughout the experiment (relative abundance 34.0 ± 4.4%; Fig. 4a). The other phyla, including *Spirochaetota*, *Cloacimonadota* and *Desulfobacterota* were less abundant, representing 14.8 ± 2.9%, 10.7 ± 1.8%, 7.1 ± 0.6% of the community, respectively. The archaeal community was dominated at the genus level by *Methanomassiliicoccus* (31.2 ± 6.3%), *Candidatus* Methanofastidiosum, *Methanosaeta* and members of the *Bathyarchaeia* family (Fig. 4c). The ecological indices, including richness, diversity and evenness remained quite stable over time for the bacterial community (Fig. 5a–c). Evenness and diversity increased over time for archaea (Fig. 5d–f), most probably due to a decrease in the abundance of *Methanomassiliicoccus* after week 6 (Fig. 4c). The dominant bacterial and archaeal groups present in the LNR are typically found in uninhibited AD microbiomes [29] and were identified in our laboratory reactors during previous experiments performed with a similar inoculum [30].

### Initial process status and microbiome of the FAN-intoxicated reactors

At day 0, crucial AD metabolic pathways were inhibited in all HNRs. First, hydrolysis was inefficient as attested both visually by the accumulation of raw sugar beet pulp residue in the slurry, and analytically by the high total solid (TS) content (Fig. 2f and Table 2) and the absence of detectable CO_2_ production (Table 2). However, products of hydrolysis were still being generated from sugar beet pulp degradation, probably at a suboptimal rate. Indeed, acidogenesis remained active, as indicated by the TVFA accumulation observed in all HNRs during the last week of the FAN intoxication experiment [24]. Second, the syntrophic oxidation of propionate and butyrate to acetate (i.e., heterotrophic acetogenesis) was inhibited, as these VFAs largely accumulated in the slurry. Third, acetoclastic methanogenesis pathway was also inhibited, as suggested by the absence of CH_4_ production (Table 2), and the high acetate concentration in the slurry. Fourth, hydrogenotrophic methanogenesis pathway was halted, since both total inorganic carbon (TIC) (Fig. 2e) and H_2_ (Fig. 4c, e, g) were available in the slurry, while no CH_4_ was produced.

**Table 2:** Process status of the reactors before initiating the process recovery strategy (day 0)

Process stability indicator	LNR	HNR1	HNR2	HNR3
pH	7.11	8.36	8.32	8.30
TAN (g_N L^−1^)	0.56	17.92	18.20	17.64
FAN (g_N L^−1^)	0.01	4.07	3.84	3.59
TS (g kg^−1^)	22.5	27.2	29.90	30.00
TVFA (g kg^−1^)	0.03	21.99	21.99	23.63
Acetate (g kg^−1^)	0.02	17.68	17.49	18.91
Propionate (g kg^−1^)	0.01	3.34	3.46	3.73
Biogas production (L_N_ L^−1^ week^−1^)	6.52	ND	ND	ND

*FAN* free ammonia nitrogen, *HNR1,2,3* free ammonia intoxicated reactors, *LNR* low nitrogen input reactor used as a reference reactor, *L*_*N*_ normalized litre of gas, *ND* not detected, *TAN* total ammonia nitrogen, *TS* total solids, *TVFA* total volatile fatty acids.

An acute inhibition of the AD process was promoted by the extremely high FAN and the TVFA concentrations in the slurry of the HNRs at the end of the FAN intoxication period (Table 2). The phenomenon of a critical FAN intoxication has not been previously examined and in general, mostly reports on moderated FAN concentration can be found in the literature. For example, Fernandes et al. [31] observed that ammonia did not significantly affect the hydrolysis rate in mesophilic reactors exposed to FAN concentrations ranging from 0.28 to 0.96 g_N L^−1^. In contrast, our study indicates that a hydrolysis inhibition will occur in mesophilic conditions when the reactors are exposed to FAN concentration higher than 3.5 g_N L^−1^.

The initial abiotic parameters of the HNRs differed from those of the reference LNR (Figs. 2, 3). Accordingly, the initial microbiome structure of the HNRs varied substantially (Fig. 4). The overall bacterial richness was lower than in the control LNR, while diversity and evenness differed greatly between the HNRs and were in the range of the LNR (Fig. 5). Members of the *Firmicutes* phylum*,* especially those belonging to the *Clostridia* order, largely dominated the HNRs. In line with our results, high FAN contents have previously been shown to select *Firmicutes* in full-scale anaerobic digesters treating cattle or swine manure [32]. At day 0, the archaeal operational taxonomic unit (OTU) 1, assigned to the *Methanosarcina* genus (100% sequence identity, Additional file 2: Table S2), accounted on average for 80.4 ± 6.6% of the relative archaeal community abundance for the HNRs (Fig. 4f, i, l). *Methanosarcina* species are versatile methanogens that can produce CH_4_ through acetoclastic, hydrogenotrophic and methylotrophic pathways [33, 34]. Lü et al. [35] observed that *Methanosarcinaceae* might shift their methanogenic pathway from acetoclastic to hydrogenotrophic as the concentrations of TAN and acetate increase. However, when facing extreme concentrations of these molecules, these authors reported that *Methanosarcinaceae* could not survive even functioning by hydrogenotrophic methanogenesis and would then be replaced by other hydrogenotrophic methanogens. At day 0, strictly hydrogenotrophic archaeal genera (*Methanobacterium*, *Methanoculleus*) were minimal in the HNRs, which suggests that this phenomenon did not occur during the intoxication experiment. Interestingly, De Vrieze et al. [33] linked the resistance of *Methanosarcina* sp. to high TAN concentrations to their ability to grow in clusters, contrarily to other methanogens. At day 0, strictly acetoclastic methanogens of the *Methanosaeta* genus were almost absent in the microbiome of the HNRs (Fig. 4f, i, l), while they were abundant in the LNR (Fig. 4c).

### Impact of the reconditioning stage on the FAN-intoxicated reactors

The initial three-phase reconditioning stage (Table 1) had a major impact on both the TAN and the FAN content in the slurry. As a result of the process reconditioning stage—phase I (pH neutralization with acetate), the increased acetate concentration in the reactor (Fig. 2d) caused the pH drop to 7.6 ± 0.0 in the three HNRs (Fig. 2b). This pH value remained higher than the one measured for the LNR (pH ~ 7.2). However, since our process recovery strategy aimed at re-establishing both acetoclastic and hydrogenotrophic methanogenesis in our HNRs, we chose to limit the initial acetate introduction to a reasonable amount. Indeed, when combined to high TAN concentration, high acetate concentration was previously identified as a factor promoting syntrophic acetate oxidation coupled to hydrogenotrophic methanogenesis in AD reactors [35]. Nevertheless, such a limited pH drop was sufficient to cause a sharp reduction in the average FAN content in the HNRs (Fig. 2c), from 3.8 ± 0.2 to 0.9 ± 0.1 g_N L^−1^. Subsequently, during the process reconditioning stage—phase II (water dilution only) and the process reconditioning stage—phase III (water dilution and re-inoculation with the LNR effluents), the average TAN content in the HNRs decreased from 17.9 ± 0.3 (week 1) to 11.7 ± 0.4 g_N L^−1^ (week 4), corresponding to an average concentration drop of 35%. During these two dilution phases, the FAN content did not undergo any further changes for HNR2 (Fig. 2c), due to a pH increase (Fig. 2b). In contrast, the FAN content clearly dropped for HNRs 1 and 3 (Fig. 2c). Overall, the three-phase reconditioning stage allowed the average FAN content in the HNRs to be reduced to 0.67 ± 0.27 g_N L^−1^ (week 4), corresponding to a total concentration drop of ~ 82%. It has been reported that a similar final value of the FAN content (~ 1 g_N L^−1^) allowed the biogas production to restart in a mesophilic continuously stirred tank reactor (CSTR) fed with chicken manure recovering from critical FAN intoxication [28].

At the phylum level for bacteria and at the genus level for archaea, the three-phase reconditioning stage did not drastically alter the microbial composition of the HNRs (Fig. 4), which remained very different from the LNR. *Methanosarcina* and *Firmicutes* remained dominant in the archaeal and bacterial communities, respectively. However, clearer changes in the microbiome of the HNRs were detected at the OTU level (Fig. 4e, h, k), as attested by the dynamics of the ecological indices (Fig. 5). During the reconditioning stage – phases II and III, a potential washout of the microbial community was expected with the addition of water. However, the bacterial evenness and diversity indices did not change significantly in HNRs 1 and 2 and remained similar to those determined for the LNR; they decreased only for HNR3 (Fig. 5b, c). During the reconditioning stage – phase III, supplying the HNRs with LNR effluents increased the archaeal richness for all the HNRs, indicating that the number of distinct archaeal OTUs increased in the slurry of the HNRs. The archaeal community of the HNRs also became slightly more even (Fig. 5e), mostly due to the increased abundance of OTU_2, assigned to the *Methanomassiliicoccus* genus (100% sequence identity, Additional file 2: Table S2) and the progressive disappearance of OTU_1, assigned to *Methanosarcina*. Although the resilience of an AD microbiome cannot be assessed solely on the basis of ecological parameters [36, 37], we interpreted the increase of the archaeal richness and evenness as a sign that the reconditioning stage of our strategy made the environmental conditions in the HNRs more favourable to the re-establishment of a functional methanogenesis. Similarly, the bacterial richness increased in all HNRs during the reconditioning stage – phase III (Fig. 5a); however, this tendency was slightly less marked for HNR3. This observation may indicate that bacterial OTUs from the LNR established in the microbiome of the HNRs are potentially capable of hydrolysis. However, the reconditioning stage mostly resulted in the increased abundance of the bacterial OTU_13, especially in HNRs 2 and 3 (Fig. 4h, k). This microbe, assigned to the *Caldicoprobacter* genus (80% sequence identity; Additional file 2: Table S1), was not abundant in the LNR slurry (maximal relative abundance of 0.01%). *Caldicoprobacter* were shown to dominate the bacterial community of mesophilic AD reactors at TAN concentrations higher than 10 g_N L^−1^ [38], which is similar to the TAN content remaining in slurry at the end of the reconditioning stage. This means that these bacteria are well-adapted to high TAN concentrations and might thus be good indicators of increasing N content in the reactor’s slurry.

During the process reconditioning stage—Phase I, the three HNRs temporarily released high volumes of CO_2_ and H_2_S (Fig. 3), probably due to the pH drop caused by the acetate addition. However, none of the HNRs produced biogas during the process reconditioning stage—Phases II and III (Fig. 2g), despite the high carbon content potentially convertible to CH_4_ and CO_2_ and available in their slurry in the form of acetate and TIC (Fig. 2d, e). Therefore, such an absence of biogas production suggests that both the acetoclastic and the hydrogenotrophic methanogenesis pathways remained inhibited in the HNRs at the end of the reconditioning stage, despite the reduction of the FAN and TAN content and the reactor re-inoculation with LNR effluents. Nielsen and Angelidaki [27] compared different dilution strategies to restore the AD process in FAN-intoxicated thermophilic reactors that stopped producing CH_4_. In their study, the reactors were exposed to a FAN concentration of 1.2 g_N L^−1^ for only 5 days before the dilution strategies were applied. In contrast, our HNRs were exposed to FAN concentrations higher than 2 g_N L^−1^ for more than 2 weeks (maximal FAN concentration > 3.5 g_N L^−1^) in a phase preceding our process recovery strategy [24]. Therefore, these extreme FAN concentrations could explain the prolonged inhibition of the AD process observed in our HNRs during the reconditioning stage of our recovery strategy.

### Restoration of CH_4_ production for the FAN-intoxicated reactors

**Fig. 6:**
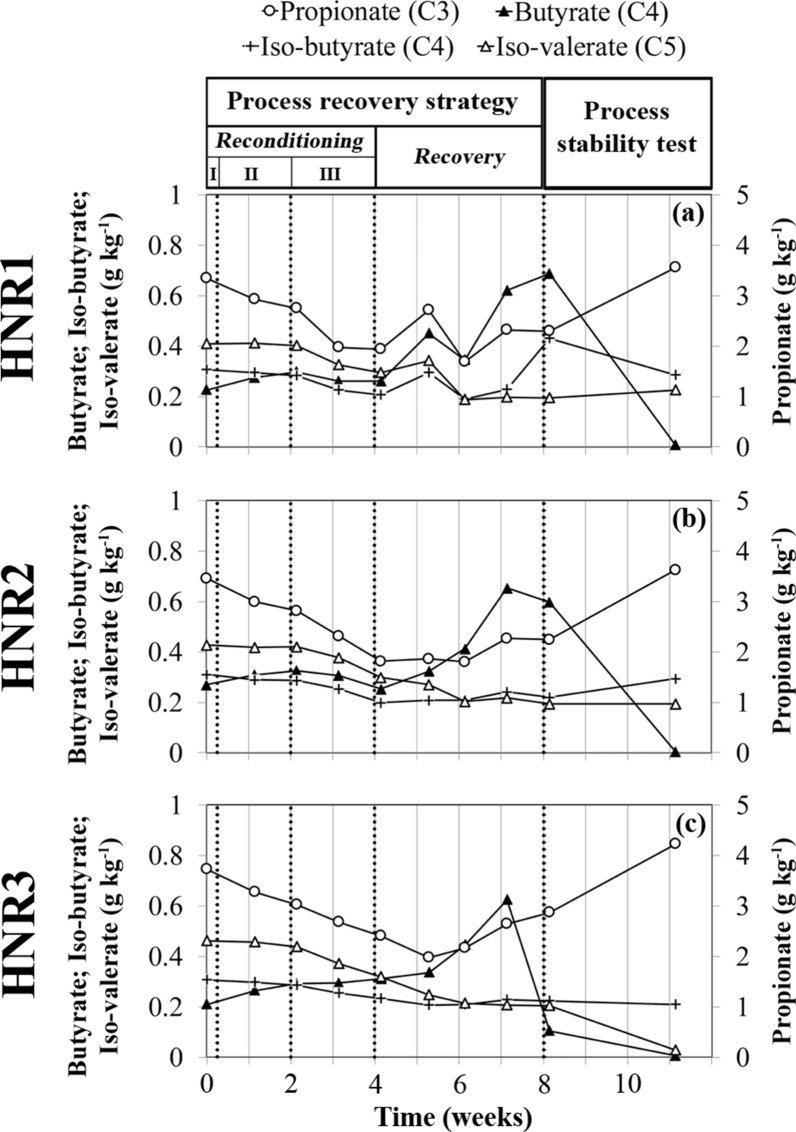
Progress over time of the concentration of C3 to C5 volatile fatty acids in the slurry for: **a** the free ammonia intoxicated reactor, HNR1, **b** HNR2 and **c** HNR3. The valerate (C5) concentration was below the detection limit of the analytical method during the whole experiment. “Process reconditioning I, II, III”, “Process recovery” and “Process stability test” refer to the phases described in Table 1

**Fig. 7:**
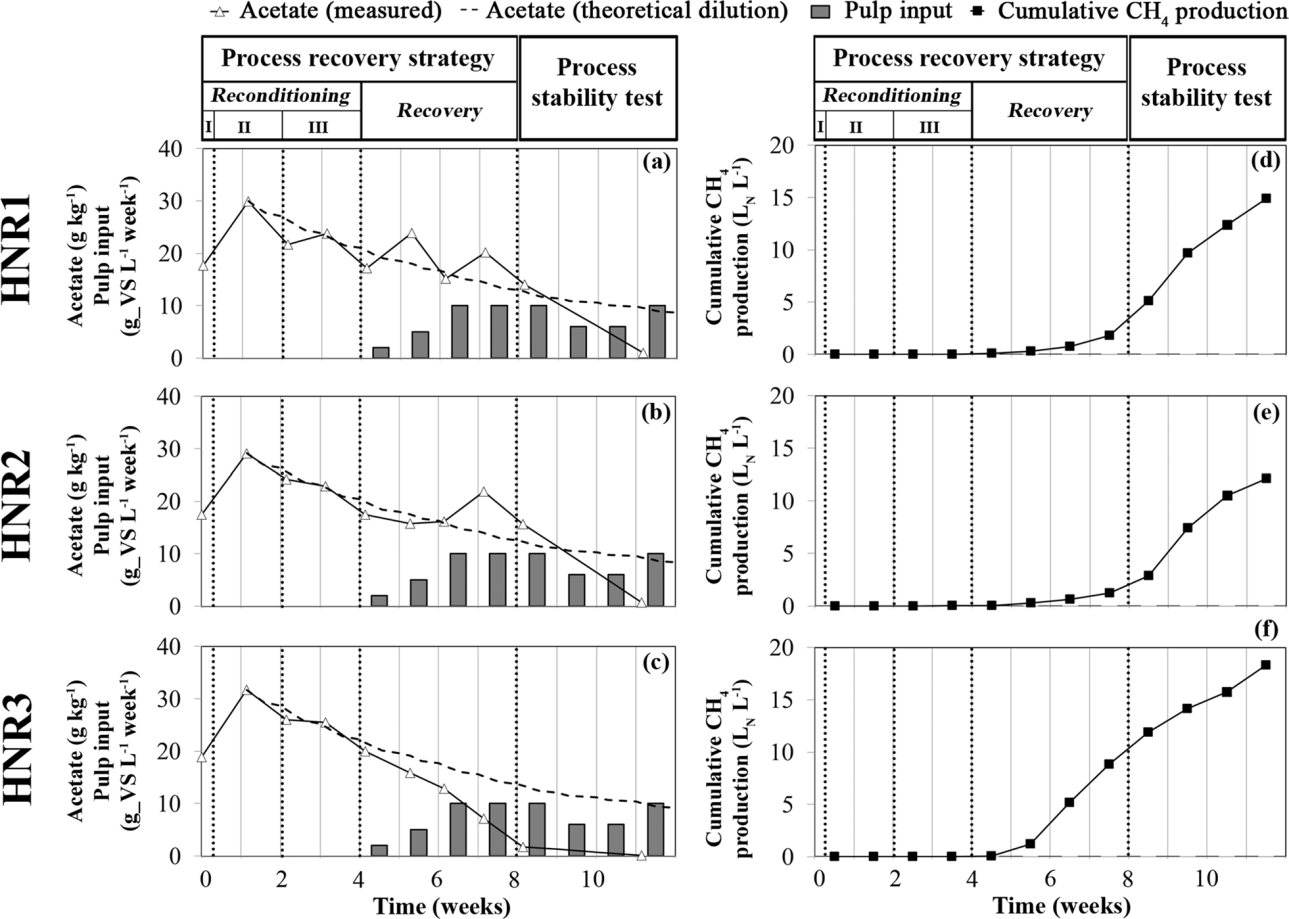
Comparison of the acetate concentration measured in the slurry and the acetate concentration expected when considering that wash-out is the only mechanism of removal (absence of microbial synthesis or degradation) for: **a** the free ammonia intoxicated reactor, HNR1, **b** HNR2 and **c** HNR3. The weekly pulp input is also presented for each HNR. Cumulative CH_4_ production for: **d** HNR1, **e** HNR2 and **f** HNR3. “Process reconditioning I, II, III”, “Process recovery” and “Process stability test” refer to the stages and phases described in Table 1

To verify whether hydrolysis remained inhibited and to re-initiate the acetoclastic and hydrogenotrophic methanogenesis pathways, the HNRs were subsequently fed with a complex organic substrate in the form of sugar beet pulp (process recovery stage, Table 1). The OLR of the HNRs was progressively increased (Fig. 2g), while the introduction of water and effluents from the LNR reference reactor remained the same. During the first week of this treatment (OLR_HNRs_ = 0.5 g_VS L^−1^ d_feed^−1^), the TAN and FAN contents further decreased in the slurry (Fig. 4a, c). Interestingly, the HNRs restarted biogas production (Fig. 2g), although it remained very low during week 4. This observation became much clearer for all the HNRs during week 5 (OLR_HNRs_ = 1 g_VS L^−1^ d_feed^−1^). From week 5 onwards, the behaviour of HNR3 diverged from that of HNRs 1 and 2. HNRs 1 and 2 initially produced little biogas (Fig. 2g) (weeks 5–6) but with a high CO_2_ content, which finally decreased during week 7 in favour of CH_4_ (Fig. 3c, e). For these two reactors, the pH of the slurry slightly decreased (Fig. 2b), while H_2_S was released into the biogas (Fig. 3d, f). Concomitantly, the TS content in the slurry stopped decreasing compared to the process reconditioning stage and subsequently did not progress much despite the daily supply of sugar beet pulp (Fig. 2f), suggesting a progressive improvement of the hydrolysis for HNRs 1 and 2. The faster synthesis of acetate (Fig. 2d), butyrate and propionate (Fig. 6a, b) in the slurry also confirms that products of hydrolysis (i.e., feedstocks of the acidogenesis stage) were synthesised at a higher rate than previously. During weeks 5–6, the H_2_ concentration in the biogas decreased (Fig. 3d, f), suggesting microbial consumption of H_2_. This could indicate the progressive restoration of the hydrogenotrophic pathway and/or a potential contribution of the H_2_-dependent methylotrophic pathway to CH_4_ production, which is consistent with the microbiome dynamics in the HNRs (see below). Indeed, the archaea involved in both pathways compete for H_2_ as a common substrate [39]. On the other hand, the neat synthesis of acetate (Fig. 7) and the low CH_4_ production compared to HNR3 (Figs. 2g and 3c, e), while acetate was abundant in the slurry (Fig. 2d), suggest that the acetoclastic pathway remained inhibited. In contrast, for HNR3, CH_4_ was produced massively from week 5 to week 7 (Figs. 2g and 3g), while the acetate concentration in the slurry decreased from 15.9 to 1.7 g kg^−1^ (Fig. 2d). The dilution rate of the slurry cannot explain such an important concentration drop (Fig. 7c), which suggests the microbial consumption of acetate and advocates for the restoration of the acetoclastic pathway in HNR3. However, the TS accumulated concomitantly in the slurry (Fig. 2f), suggesting that the hydrolysis remained suboptimal in HNR3 at the end of the process recovery stage. We attributed this prolonged inhibition of the hydrolysis to the pH increase (pH range of 7.7–8.0; Fig. 2b) that was caused by the microbial consumption of acetate. Indeed, Romsaiyud et al. [40] showed that the optimal pH range for efficient lignocellulosic biomass hydrolysis was between 2.6 and 7.5 at 37°C.

### Microbial community dynamics in the FAN-intoxicated reactors during the restoration of the CH_4_ production

Improved hydrolysis for HNRs 1 and 2 (week 5) coincided with an abrupt decrease of the bacterial richness, evenness and diversity (Fig. 5a–c), while the archaeal ecological indices transiently increased (Fig. 5d–f). The same indices reminded relatively stable for HNR3. The change in the bacterial community structure was related to an increased relative abundance of the *Firmicutes* phylum and a decreased relative abundance of the *Bacteroidota* and *Spirochaetes* phyla (Fig. 4d, g). Commonly described butyrate-producing bacteria belong to the *Firmicutes* phylum and the *Clostridia* order [41], which could explain the rising butyrate concentration in all HNRs (Fig. 6). The change in the archaeal richness, evenness and diversity reflected a drastic reshaping of the community structure, due to the replacement of the formerly dominant archaeal OTU_1 (assigned to the *Methanosarcina* genus; Additional file 2, Table S2) by OTU_4 (assigned to the *Candidatus* Methanoplasma genus) in HNRs 1 and 2. The latter OTU reached more than 50% of the relative archaeal abundance (weeks 5–6, Fig. 4f, i). In contrast to HNRs 1 and 2, the dominance of a versatile *Methanosarcina* coincided with an increased acetate consumption in HNR3, which may indicate the restoration of the acetoclastic pathway in this reactor.

Members of the *Candidatus* Methanoplasma genus are strictly methylotrophic methanogens that can only produce CH_4_ from the hydrogen-dependent reduction of methanol or methylamines [42]. In a recent study [39], *Candidatus* Methanoplasma were shown to play a key role in the successful bio-augmentation of mesophilic reactors operating at high FAN concentrations. Therefore, their rise in abundance suggests that the methylotrophic pathway could have contributed to the restoration of the CH_4_ production in HNRs 1 and 2, possibly in combination with the hydrogenotrophic pathway, while the acetoclastic pathways were of minor importance at that stage.

We could further link the progressive dominance of *Candidatus* Methanoplasma and the improvement of the hydrolysis in HNRs 1 and 2 via the canonical correspondence analysis (CCA) computed for the archaeal genera (Additional file 1: Fig. S2). It highlighted a negative corelation between the relative abundance of Candidatus *Methanoplasma* and the TS content in the slurry of the HNRs. Tian et al. [43] also observed the appearance of strictly methylotrophic methanogens in exposing mesophilic inoculums at increasing FAN concentrations (up to ~ 1 g_N L^−1^). The authors linked this phenomenon to the presence of bacteria assigned to the *Tissierella* genus. Indeed, these microbes can produce methylamine from different amino acids and could, therefore, provide methylotrophic methanogens with substrate. In our reactors, only the bacterial OTU_47 was assigned to an unclassified member of the *Peptostreptococcales–Tissierellales* family (100% sequence identity; Additional file 2: Table S1). Interestingly, in week 5, the relative abundance of this bacteria was higher in HNR1 (1.31%) and HNR2 (1.61%) than in HNR3 (0.67%). Therefore, the improvement of the sugar beet pulp hydrolysis in HNRs 1 and 2 may have induced the release of amino acids in the slurry, which in turn were converted to methylamines by this *Peptostreptococcales–Tissierellales* member, generating favourable conditions for the preferential growth of *Candidatus*_Methanoplasma.

The resumed provision of sugar beet pulp also resulted in the increased abundance of *Methanoculleus* in all HNRs (Fig. 4f, i, l). *Methanoculleus* sp. are strictly hydrogenotrophic methanogens [44] and were reported to outcompete *Methanosaeta* sp. at higher OLR (> 1 g_COD L^−1^ day^−1^) [45, 46], which could explain their sudden appearance. This could indicate a progressive restoration of the hydrogenotrophic methanogenesis pathway in the HNRs. It should be noted that the contribution of putative SAO to the CH_4_ production of the HNRs was most likely very low. Their total relative abundance in the slurry of the HNRs remained < 2% during the whole experiment. Such low abundance is in line with the fact that acetoclastic methanogenesis has a thermodynamic advantage over SAO-induced hydrogenotrophic methanogenesis at mesophilic temperatures [47]. Nevertheless, the presence of other as yet unknown SAO cannot be excluded.

### Testing the process stability in the FAN-intoxicated reactors after the process recovery strategy

To assess the operational stability of the recovered HNRs, the re-inoculation was interrupted from week 8 onwards, while sugar beet pulp feeding was maintained according to the feeding scheme applied to the LNR (process stability test stage, Table 1). For all HNRs, propionate started to accumulate in the slurry after the re-inoculation was interrupted (Fig. 6, weeks 8–11), which may be associated with the gradual decline of syntrophic propionate oxidizers (SPO) belonging to the *Cloacimonadota* bacterial phylum (Fig. 4d, g, j) [48]. Indeed, in our reactors, only *Cloacimonadota* members were identified as potential SPOs and they were more abundant in the LNR effluents (Fig. 4a) than in the slurry of the HNRs. This indicates that re-inoculation was a key element of our process recovery strategy in stabilizing the AD process of the HNRs during the restoration of their CH_4_ production.

During the process stability test, the biogas production dynamics of the HNRs resembled those of the LNR, except during the last week of the experiment for HNR2 (Fig. 2g), where a slight decrease in biogas production was accompanied by the accumulation of TS in the slurry (Fig. 2f). Otherwise, the TS content in the slurry of HNRs 1 and 2 remained constant, suggesting that the hydrolysis of the fed sugar beet pulp was preserved in these reactors. HNRs 1 and 2 maintained a similar [CH_4_]/[CO_2_] ratio until the end of the experiment (Fig. 3c, e), which was substantially higher during weeks 8–9 (Fig. 2g) due to a rise in pH (Fig. 2b) that favoured the CO_2_ capture in the slurry. This pH rise was probably caused by the depletion of acetate that reached a negligible concentration at the end of the experiment (Fig. 2d). The dilution rate of the slurry could not explain such an important acetate concentration drop (Fig. 7c), and, therefore, suggests its microbial consumption and advocates for the restoration of the acetoclastic methanogenesis in HNRs 1 and 2. During the last week of the experiment, the CO_2_ content in the biogas produced by HNRs 1 and 2 transiently increased (Fig. 3c, e). However, as CH_4_ production continued, we attributed this phenomenon to the final depletion of acetate, followed by a fluent transition to CH_4_ production from the fed sugar beet pulp only. For HNR3, the [CH_4_]/[CO_2_] ratio in the biogas remained close to 1 and its dynamics adopted a weekly pattern analogous to that of the LNR (Fig. 3g). Concomitantly, TS stopped accumulating in the slurry (Fig. 2f), suggesting an improvement of the pulp hydrolysis. In HNR3, CH_4_ was continuously produced (Fig. 7f), while the residual acetate concentration in the slurry was negligible (Fig. 7c), suggesting that the sugar beet pulp degradation was the main source of CH_4_ production. During the last week of the experiment, the average pulp to CH_4_ conversion ratio was 0.41 ± 0.10 L_N__CH_4_ g_VS^−1^ for the HNRs, which is analogous to the pulp BMP (0.38 ± 0.03 L_N__CH_4_ g_VS^−1^). Therefore, we conclude that the AD process was successfully restored for the three HNRs. The pH of the slurry as well as its TAN and FAN content were similar for all HNRs (Fig. 2a–c) and approached the values observed for the LNR. However, the AD process remained slightly perturbed in the HNRs as evidenced by propionate accumulation, which is attributable to the fact that re-inoculation was interrupted too early to ensure the long-term process stability of the HNRs.

### Final microbiome structure of the recovered reactors

The final microbiome of the HNRs remained different to that of the LNR (Fig. 4). In particular, strictly acetoclastic methanogens of the *Methanosaeta* genus remained much less abundant in the HNRs (Fig. 4f, i, l) compared to the LNR (Fig. 4c). In addition, the final values of the richness, evenness and diversity indices were lower for the HNRs than for the LNR (Fig. 5), both for bacteria and archaea. It is likely that when a stable environment is exposed to a disturbance, it changes to a deterministic one, and the better adapted competitors start to dominate, which is usually reflected by a decreased richness and diversity [37].

At the end of the experiment, *Firmicutes* still dominated the microbiome of the HNRs, but the relative abundance of *Bacteroidota* had significantly increased (Fig. 4d, g, j). A recent meta-regression study [49] comprising 846 mesophilic and 246 thermophilic AD processes revealed that these two phyla significantly determined the CH_4_ yield in both stable and non-stable process conditions. Towards the end of the experiment in HNRs 1 and 2, the relative abundance of the archaeal OTU_4, assigned to the strictly methylotrophic genus *Candidatus* Methanoplasma, was decreasing in favour of OTU_1, assigned to *Methanosarcina* (Fig. 4f, i). This could suggest an increasing contribution of the acetoclastic and hydrogenotrophic pathways to CH_4_ production, and the minor importance of methylotrophic methanogenesis in the recovered reactors. For HNR3, no large changes were observed at the archaea genus level during the stability test (Fig. 4l), and the final archaeal community remained dominated by OTU_1. In other words, the final archaeal microbiome was dominated by the same *Methanosarcina* member as on day 0 despite the re-inoculation of the HNRs with LNR effluents. Previously, efficient CH_4_ production has been achieved in *Methanosarcina*-dominated AD reactors operating at FAN concentrations up to 0.6 g_N L^−1^ [15]. The persistence of this versatile methanogen in the HNRs after the restoration of a functional AD process confirms our assumption that microbes capable of acetoclastic and/or hydrogenotrophic methanogenesis remained present in the slurry after the intoxication experiment and shows that our process recovery strategy allowed environmental conditions favourable to their activity to be restored.

### Perspectives for process recovery in real-scale biogas production plants

Due to the current price of acetic acid on the European market (December 2022), applying our strategy to ammonia intoxicated real-scale digesters of the size of several thousand m^3^ could represent a significant cost for plant managers. Indeed, it would cost US $17.5 to treat 1 m^3^ of slurry [50], which is not negligible. However, Fig. 7 shows that CH_4_ production from the added acetic acid could partially compensate the cost of its addition to real-scale FAN-intoxicated digesters. Indeed, the potential CH_4_ production from acetoclastic methanogenesis in HNRs 1 and 2 during the process recovery stage accounted for 34.4 and 48.7% of their CH_4_ production, respectively. For HNR3, the potential CH_4_ production from acetoclastic methanogenesis during the process stability test stage accounted for 77.6% of its CH_4_ production.

The initial pH drop due to the introduction of acetic acid in the slurry of the HNRs induced a quick conversion of free ammonia, a highly volatile compound causing threats to the environment and human health [51], to ammonium, a non-volatile form of nitrogen that could be used as an interesting fertilizer [52]. In addition, acetic acid has recently been proposed as a low-cost biostimulant under drought stress for a diverse range of major crops, such as maize [53], cassava [54] and mung bean [55]. Therefore, our process recovery strategy could allow the open-air storage and field application of the effluents produced by real-scale digesters recovering from FAN intoxication. Such agronomic valorization of the reactors’ effluents could be more problematic with alternative dilution strategies that do not involve an initial pH adjustment of the slurry [27, 28]. These benefits are an additional compensation for the high cost of acetic acid and relativize the long duration required to reach full process recovery after applying our strategy.

Re-inoculating real-scale digesters exposed to ammonia intoxication could be regarded as challenging, in the sense that it would require the provision of large quantities of “healthy” digestate. We would not advise biogas plant managers to use a post-digester as a source of uninhibited biomass as it is most likely exposed to the same TAN concentration that the intoxicated main digester. Therefore, daily transportation of fresh digestate from the closest uninhibited biogas plant could be necessary to ensure the stability of the process during the process recovery period. Applying our strategy to a typical farm-scale digester of 1000 m^3^ working volume would require transporting 6.5 m^3^ of fresh digestate per day during the re-inoculation period, which is feasible with a slurry tanker. In our opinion, applying such a complex strategy could be justified compared to a complete restart of the process. Indeed, we showed in a previous work [56] that establishing a functional AD process during the start-up phase of a full-scale mesophilic digester fed with psychrophilic substrates required > 120 days. In addition, the microbiome resulting from such a start-up phase was not acclimatized to high FAN concentrations, whereas the microbiome of a digester that has recovered from ammonia intoxication could appear more resilient to future intoxication events.

## Conclusions

This study describes the AD process recovery strategy of three anaerobic reactors that were previously exposed to a complete process collapse caused by extreme free ammonia intoxication. The initial reconditioning stage of the process recovery strategy, which successively included (i) pH reduction by acetic acid, (ii) water dilution and (iii) re-inoculation combined with moderate water dilution, did not allow the biogas production to be re-established. Restored feeding with sugar beet pulp after the reconditioning stage appeared to be decisive for the re-establishment of biogas production, while re-inoculating the reactors appeared to be essential to maintain process stability during the process recovery period. Overall, the proposed process recovery strategy was successful in restoring an efficient conversion of the substrate fed to CH_4_ for the three intoxicated reactors within 11 weeks. Furthermore, the initial introduction of acetic acid allowed substantial CH_4_ production during the recovery period. Finally, introducing acetic acid in the intoxicated reactors promoted ammonium sequestration in the slurry. This could enable the open-air storage and field application of the effluents produced by full-scale biogas production digesters recovering from FAN intoxication. However, to ensure the long-lasting process recovery of such digesters, we recommend prolongating the re-inoculation over a longer period than assessed here. Additional lab-scale experiments integrating reactors that are not re-inoculated could allow the impact of re-inoculation on process stability to be better assessed. In the re-inoculated reactors, the introduction of uninhibited effluents should be prolongated until the propionate concentration drops. Finally, the reactors that recovered from FAN-intoxication should be compared with reference reactors (treated like the LNR) in a post-recovery phase during which all reactors would be fed with nitrogen-rich substrates. This final phase could allow us to verify whether the recovered reactors developed a higher tolerance to FAN or not. Finally, the present study highlights the importance of replicates when working with (semi-)continuously fed AD reactors exposed to stress. Indeed, although the three intoxicated reactors had been exposed to the same treatment from their inoculation, they showed contrasting behaviours and distinct microbiome dynamics during the experiment.

## Materials and methods

### FAN intoxication

The method used to generate severe FAN intoxication in the reactors is described in detail in Lemaigre et al. [24]. The experiment was performed in mesophilic temperature conditions (37 ± 0.5 °C), using four stainless steel tank reactors of 100L working volume, continuously stirred at 60 rotations per minute (rpm) with a central anchor-type stirrer and presenting an additional 25L headspace volume. All four reactors were initially and simultaneously inoculated with anaerobic sludge from a mesophilic anaerobic reactor at a wastewater treatment plant (Schifflange, Luxembourg). Then, the reactors were manually fed with commercial dried sugar beet pulp pellets used as the basal diet (Additional file 1: Table S2) on working days, usually 5 consecutive days per week (d_feed), following a semi-continuous scheme. The pulp contained 0.012 gram of nitrogen per gram of volatile solids (g_N g_VS^−1^) and its biochemical methane potential (BMP) was measured to be 0.38 normalized litres of CH_4_ per gram of VS (L_N_ CH_4_ g_VS^−1^) according to standard BMP assessment methods [57]. The pulp organic loading rate (OLR) was 2 g of VS per litre of slurry and per feeding day (g_VS L^−1^ d_feed^−1^), i.e., 1.4 g_VS L^−1^ day^−1^ on a weekly average, while the hydraulic retention time (HRT) was adjusted at 56 days by adding tap water at 37°C to the pulp feed. The four reactors were initially acclimatized to the substrate for 8 weeks, then three reactors (referred to as high nitrogen input reactors, with the respective acronyms HNR1, HNR2 and HNR3) underwent identical exposure to increasing nitrogen input by adding urea to the basal sugar beet pulp diet (maximal nitrogen supply of 0.95 g_N L^−1^ d_feed^−1^). After 23 weeks, this treatment led to severely FAN-intoxicated reactors, with a process collapse marked by the complete interruption of biogas production due to high pH and FAN content (pH > 8.3 and FAN > 3.5 g_N L^−1^; Table 2). The fourth reactor, utilized as a reference (low nitrogen input reactor, LNR), was maintained in a steady state during the whole experimental period with a low nitrogen supply using the abovementioned feeding regime based only on sugar beet pulp pellets. Following its inoculation, the LNR efficiently converted the fed substrate to CH_4_ with a yield of 0.33 ± 0.03 L_N_ CH_4_ g_VS^−1^, which remained close to the pulp BMP.

After 23 weeks of gradual intoxication, the feeding of the three FAN-intoxicated reactors (HNRs) was stopped, and these reactors were left to rest for 3 days before initiating the process recovery strategy described below. During this resting period, no biogas production was detected for the HNRs.

### Process recovery strategy and assessment of the process stability during post-intoxication recovery

The process recovery strategy involved a process reconditioning stage followed by a process recovery stage (Table 1 and Fig. 1) and was applied in an identical way to the three FAN-intoxicated reactors (HNRs), while the treatment applied to the reference reactor (LNR) during the 23-week FAN-intoxication period was left unchanged (i.e., sugar beet pulp as the unique substrate; OLR and HRT set to 2 g_VS L^−1^ d_feed^−1^ and 56 days, respectively). As a follow-up, a process stability test stage was performed to assess the process recovery of the HNRs. The organization of the process recovery strategy is detailed hereafter.

*Process reconditioning stage—Phase I: pH neutralization with acetic acid* (day 0). First, a composite sample (1:1:1) of slurries from the three HNRs was titrated against pure acetic acid (Sigma-Aldrich, USA) until the pH was reduced from 8.3 to 7.7, which required the injection of 15 g_VS of acetic acid per litre of slurry. Then, this amount of pure acetic acid was injected into the slurry of the HNRs at a flow rate of 0.03 g_VS per litre of slurry and per minute using a peristaltic pump (IPC 8, Ismatec, Germany).

*Process reconditioning stage—Phase II: Water dilution* (end of week 0 and week 1). For each HNR, 25 mL of tap water at 37°C was introduced per litre of slurry and per feeding day (mL L^−1^ d_feed^−1^), according to a 56-day HRT.

*Process reconditioning stage—Phase III: Water dilution and re-inoculation* (week 2 to week 3). Each HNR was fed with 6.5 mL LNR effluents per litre of slurry and per feeding day. The LNR effluents were supplemented with tap water at 37°C to reach a total volume of 25 mL L^−1^ d_feed^−1^, according to a 56-day HRT.

*Process recovery stage: water dilution, re-inoculation and feeding* (week 4 to week 7). For each HNR, sugar beet pulp was introduced following a progressively increasing OLR to reach a value of 2 g_VS L^−1^ d_feed^−1^ (from week 6 onwards), identical to the steady OLR applied to the LNR. The introduction of effluents produced by the LNR into the three HNRs was maintained (6.5 mL L^−1^ d_feed^−1^). The feed was supplemented with tap water at 37°C to reach a total volume of 25 mL L^−1^ d_feed^−1^, according to a 56-day HRT.

*Process stability test stage: Feeding and water dilution* (from week 8 onwards). To assess the process stability after the recovery strategy, the three HNRs were fed in the same manner as the LNR, i.e., 2 g_VS sugar beet pulp L^−1^ d_feed^−1^ as the sole substrate, supplemented with tap water at 37°C to reach a total volume load of 25 mL L^−1^ d_feed^−1^, according to a 56-day HRT.

### Slurry monitoring

Every Monday, the total ammonia nitrogen content (TAN, g_N L^−1^), the total inorganic carbon content (TIC, g_CaCO_3_ L^−1^), the individual VFA concentrations (C2 to C5, g kg^−1^), the total solids content (TS, g kg^−1^) and the pH were measured in the slurry. The TAN and the TIC contents were measured using the BiogasPro system (RIMU, Königsbrunn, Germany) in conformity with the manufacturer’s protocol and as performed in a previous work [30]. For the TAN content measurement, the BiogasPro method is equivalent to the Quantofix N-Volumeter method (Terraflor, Iserlohn, Germany) that was previously described [58]. For the TIC content measurement, the BiogasPro method consists in acidifying 200 mL slurry with 150 mL HCl 5%v/v and to measure the volume of the released CO_2_, visualized by the displacement of a water column in a graduated cylinder [59, 60]. The individual VFA concentrations, the TS content and the pH of the slurry were measured according to the methods exposed in [30]. The total VFA concentration (TVFA, g kg^−1^) was expressed as the sum of the individual VFA concentrations, measured for acetate, propionate, iso-butyrate, butyrate, iso-valerate and valerate. The FAN content in the slurry (g_N L^−1^) was calculated on the basis of the TAN content and the pH [19], with a temperature of 37°C and a pKa of 8.89.

To assess the fate of the acetic acid provided for process recovery, for each HNR, the concentration of acetate measured in the slurry was compared with the concentration that could be expected in the absence of microbial activity, i.e., if the variation of the acetate concentration was only due to the dilution and wash-out (Fig. 7).

### Bacterial and archaeal community monitoring

Aliquots of about 200 µL of slurry were sampled once per week (on Mondays) throughout the experiment and for each studied reactor. Aliquots were immediately snap frozen in liquid nitrogen and stored at -80°C prior to further analysis. Total DNA was extracted using the DNeasy PowerSoil Kit from Qiagen (Carlsbad, CA) according to the manufacturer’s protocol. The 16S rRNA gene amplicon libraries were then prepared, sequenced, de-multiplexed, quality trimmed, clustered into operational taxonomic units (OTUs) at 97% similarity and taxonomically assigned using the Silva 138 database as previously explained [48]. Briefly, separate primer pairs targeting the V6–V8 and V4–V6 regions, respectively, of 16SrRNA were used to assess the bacterial and archaeal communities. All the bioinformatic processing steps were done using usearch v11 [61]. On average, 65.5% of reads passed the quality control thresholds and the resulting read counts were normalized to 10,000 reads per sample for further comparative analyses. SAO and SPO were identified using blast against the Acetobase database [62] and using the 16S rRNA gene signatures of known SPOs [63], respectively. Matches with a sequence similarity of over 97% were retained as positive for both SAOs and SPOs. The bacterial and archaeal community richness and diversity were calculated with the sobs and invsimpson calculators in mothur (v.1.34.4 or later [64]**)**, respectively. The higher these indices, the higher the richness and diversity of the community studied, respectively. Community evenness was measured via the Simpson even coefficient ranging from 0 (uneven community; one or several dominant OTUs and many singlets) to 1 (perfectly even community; all OTUs present at the same relative abundance). The influence of process parameters on the bacterial and archaeal community composition was analysed using the canonical correspondence analysis (CCA) with R and the Vegan package [65] (Additional file 1: Fig. S1 and Fig. S2, respectively). The nucleotide sequences obtained from sequencing were deposited in the GenBank database (http://www.ncbi.nlm.nih.gov/genbank/) and are part of a bigger submission with accession numbers from OQ217616 to OQ219830 for bacteria and from OQ220520 to OQ220896 for archaea.

### Biogas monitoring

The biogas produced by each reactor passed through a cooling unit at ~ 8 °C to remove excess water vapour and the dried biogas was collected in an 80-L gas bag used as storage (Tecobag, Tesseraux, Germany). The biogas measurement method is detailed in our previous study describing the FAN-intoxication period [24]. Briefly, every 2 h, the content of the bag was pumped through a recirculation loop. About 50 mL of gas was then pumped from the recirculation loop to a three-channel gas chromatograph (CompactGC, global analyser solutions™, Interscience, Belgium) to measure the CH_4_, CO_2_, H_2_, H_2_S, O_2_ and N_2_ concentrations. The GC was equipped with a thermal conductivity detector on each channel. The detectors were heated to 80 °C and the filaments to 110 °C. The channels were equipped with an RI-QBond column (10 m × 0.32 mm), Rtx-1 column (30 m × 0.32 mm) and RI-QBond pre-column (3 m × 0.32 mm) followed by a Molsieve 5A column (7 m × 0.32 mm) for the separation and analysis of the CO_2_, H_2_S and H_2_, O_2_, N_2_, CH_4_ gases, respectively. Helium and argon were used as the carrier gases for the first two and third channels, respectively. For the three channels, the elution was performed under isothermal conditions at 50 °C and with a 10 mL min^−1^ flow. The volume measurement in the gas bag was performed by a drum-type wet gas meter (TG-5, Ritter, Germany) and normalized to 0°C; 1013 hPa.

The biogas composition measurements could not be taken from week 1 to week 3 for the HNRs, due to the absence of detectable biogas production.

### Supplementary Information

**Additional file 1: Table S1.** Taxonomic affiliation of the top 50 bacterial OTUs presented in Fig. 4. **Table S2.** Composition of the dried sugar beet pulp pellets used to feed the anaerobic reactors. **Fig. S1.** Canonical correspondence analysis (CCA) ordination diplot for the top 50 more abundant bacterial OTUs. Data from all the reactors studied (the LNR and the three HNRs, 12 timepoints for each of them) were used for this analysis. Light-grey vectors represent process parameters influencing the microbial community, such as the pH (pH), volatile solids (VS), total solids (TS), total ammonia nitrogen (TAN), free ammonia nitrogen (FAN), acetic acid concentration (Acet. Ac.) and total inorganic carbon (TIC). **Fig. S2.** Canonical correspondence analysis (CCA) ordination diplot for the archaeal genera. Data from all the reactors studied (the LNR and the three HNRs, 12 timepoints for each of them) were used for this analysis. Light grey vectors represent process parameters influencing the microbial community, such as the pH (pH), total solids (TS), total ammonia nitrogen (TAN), free ammonia nitrogen (FAN), total inorganic carbon (TIC), recirculated low nitrogen reactor effluents (LNR Effluent), sugar beet pulp organic loading rate (Pulp. Input), acetic acid concentration (Acet. Ac.) and produced biogas volume (biogas).

**Additional file 2. **Relative abundance (%) and taxonomic affiliation according to Silva 138 database of 16S rRNA gene amplicon sequencing OTUs for bacteria (Table S1) and archaea (Table S2).

## Data Availability

The nucleotide sequences obtained from sequencing were deposited in the GenBank database (http://www.ncbi.nlm.nih.gov/genbank/) and are part of a bigger submission with accession numbers from OQ217616 to OQ219830 for bacteria and from OQ220520 to OQ220896 for archaea.

## Abbreviations

AD: Anaerobic digestion
BMP: Biochemical methane potential
COD: Chemical oxygen demand
CSTR: Continuously stirred tank reactor
EU: European Union
FAN: Free ammonia nitrogen
GC: Gas chromatography
HNR: High nitrogen input reactor
HRT: Hydraulic retention time
LNR: Low nitrogen input reactor
OLR: Organic loading rate
OTU: Operational taxonomic unit
SAO: Syntrophic acetate oxidizer
SPO: Syntrophic propionate oxidizer
TAN: Total ammonia nitrogen
TIC: Total inorganic carbon
TS: Total solids
TVFA: Total volatile fatty acids
VFA: Volatile fatty acid
VS: Volatile solids

## References

[1] Bórawski P, Wyszomierski R, Bełdycka-Bórawska A, Mickiewicz B, Kalinowska B, Dunn JW (2022). Development of renewable energy sources in the European union in the context of sustainable development policy. Energies.

[2] IPCC. Working Group III contribution to the Sixth Assessment Report of the Intergovernmental Panel on Climate Change. In: Core Writing Team, Jager-Waldau A, Sapkota T, editors. Climate Change 2022. Mitigation of Climate Change. Geneva, Switzerland: IPCC; 2022. p. 2913.

[3] Xue S, Zhang S, Wang Y, Wang Y, Song J, Lyu X (2022). What can we learn from the experience of European countries in biomethane industry: taking China as an example?. Renew Sustain Energy Rev.

[4] Pavičić J, Mavar KN, Brkić V, Simon K (2022). Biogas and biomethane production and usage: technology development. Advant Chall Eur Energies.

[5] Theuerl S, Herrmann C, Heiermann M, Grundmann P, Landwehr N, Kreidenweis U (2019). The future agricultural biogas plant in Germany: a vision. Energies.

[6] Ackrill R, Kay A (2014). The growth of biofuels in the 21st century: policy drivers and market challenges.

[7] EC. Commission staff working document. State of play on the sustainability of solid and gaseous biomass used for electricity, heating and cooling in the EU. Brussels; 2014.

[8] Lijó L, González-García S, Bacenetti J, Moreira MT (2017). The environmental effect of substituting energy crops for food waste as feedstock for biogas production. Energy.

[9] Yenigün O, Demirel B (2013). Ammonia inhibition in anaerobic digestion: a review. Process Biochem.

[10] Bellucci M, Borruso L, Piergiacomo F, Brusetti L, Beneduce L (2022). The effect of substituting energy crop with agricultural waste on the dynamics of bacterial communities in a two-stage anaerobic digester. Chemosphere.

[11] Jiang Y, McAdam E, Zhang Y, Heaven S, Banks C, Longhurst P (2019). Ammonia inhibition and toxicity in anaerobic digestion: a critical review. J Water Process Eng.

[12] Wijesinghe DTN, Suter HC, Scales PJ, Chen D (2021). Lignite addition during anaerobic digestion of ammonium rich swine manure enhances biogas production. J Environ Chem Eng.

[13] Yan M, Treu L, Zhu X, Tian H, Basile A, Fotidis IA (2020). Insights into ammonia adaptation and methanogenic precursor oxidation by genome-centric analysis. Environ Sci Technol.

[14] Capson-Tojo G, Rouez M, Crest M, Trably E, Steyer JP, Bernet N (2017). Kinetic study of dry anaerobic co-digestion of food waste and cardboard for methane production. Waste Manag.

[15] Capson-Tojo G, Moscoviz R, Astals S, Robles A, Steyer JP (2020). Unraveling the literature chaos around free ammonia inhibition in anaerobic digestion. Renew Sustain Energy Rev.

[16] Polag D, Heuwinkel H, Laukenmann S, Greule M, Keppler F (2013). Evidence of anaerobic syntrophic acetate oxidation in biogas batch reactors by analysis of 13C carbon isotopes. Isotopes Environ Health Stud.

[17] Conrad R (2006). Contribution of hydrogen to methane production and control of hydrogen concentrations in methanogenic soils and sediments. FEMS Microbiol Ecol.

[18] Ferry JG (1993). Methanogenesis: ecology, physiology, biochemistry and genetics (Chapman & Hall Microbiology Series).

[19] Calli B, Mertoglu B, Inanc B, Yenigun O (2005). Effects of high free ammonia concentrations on the performances of anaerobic bioreactors. Process Biochem.

[20] Westerholm M, Levén L, Schnürer A (2012). Bioaugmentation of syntrophic acetate-oxidizing culture in biogas reactors exposed to increasing levels of ammonia. Appl Environ Microbiol.

[21] Koch M, Dolfing J, Wuhrmann K, Zehnder AJB (1983). Pathways of propionate degradation by enriched methanogenic cultures. Appl Environ Microbiol.

[22] Lemaigre S, Adam G, Goux X, Noo A, De Vos B, Gerin PA (2016). Transfer of a static PCA-MSPC model from a steady-state anaerobic reactor to an independent anaerobic reactor exposed to organic overload. Chemom Intell Lab Syst.

[23] Jo Y, Cayetano RDA, Kim G-B, Park J, Kim S-H (2022). The effects of ammonia acclimation on biogas recovery and the microbial population in continuous anaerobic digestion of swine manure. Environ Res.

[24] Lemaigre S, Adam G, Gerin PA, Noo A, De Vos B, Klimek D (2018). Potential of multivariate statistical process monitoring based on the biogas composition to detect free ammonia intoxication in anaerobic reactors. Biochem Eng J.

[25] Khalil CA, Ghanimeh S, Medawar Y. Ammonia inhibition and recovery potential in anaerobic digesters: A review. In: Proceedings of the Air and Waste Management Association’s Annual Conference and Exhibition, AWMA. Pittsburgh, Pennsylvania; 2017. p. 275193.

[26] Chen Y, Cheng JJ, Creamer KS (2008). Inhibition of anaerobic digestion process: a review. Bioresour Technol.

[27] Nielsen HB, Angelidaki I (2008). Strategies for optimizing recovery of the biogas process following ammonia inhibition. Bioresour Technol.

[28] Niu Q, Qiao W, Qiang H, Hojo T, Li Y-Y (2013). Mesophilic methane fermentation of chicken manure at a wide range of ammonia concentration: stability, inhibition and recovery. Bioresour Technol.

[29] Rahman MS, Hoque MN, Puspo JA, Islam MR, Das N, Siddique MA (2021). Microbiome signature and diversity regulates the level of energy production under anaerobic condition. Sci Rep.

[30] Goux X, Calusinska M, Lemaigre S, Marynowska M, Klocke M, Udelhoven T (2015). Microbial community dynamics in replicate anaerobic digesters exposed sequentially to increasing organic loading rate, acidosis, and process recovery. Biotechnol Biofuels.

[31] Fernandes TV, Keesman KJ, Zeeman G, van Lier JB (2014). Effect of ammonia on the anaerobic hydrolysis of cellulose and tributyrin. Biomass Bioenerg.

[32] Li J, Rui J, Yao M, Zhang S, Yan X, Wang Y (2015). Substrate type and free ammonia determine bacterial community structure in full-scale mesophilic anaerobic digesters treating cattle or swine manure. Front Microbiol.

[33] De Vrieze J, Hennebel T, Boon N, Verstraete W (2012). Methanosarcina: the rediscovered methanogen for heavy duty biomethanation. Bioresour Technol.

[34] Lambie SC, Kelly WJ, Leahy SC, Li D, Reilly K, McAllister TA (2015). The complete genome sequence of the rumen methanogen Methanosarcina barkeri CM1. Stand Genomic Sci.

[35] Lü F, Hao L, Guan D, Qi Y, Shao L, He P (2013). Synergetic stress of acids and ammonium on the shift in the methanogenic pathways during thermophilic anaerobic digestion of organics. Water Res.

[36] Li L, Peng X, Wang X, Wu D (2018). Anaerobic digestion of food waste: a review focusing on process stability. Biores Technol.

[37] Goux X, Calusinska M, Lemaigre S, Marynowska M, Klocke M, Udelhoven T (2015). Microbial community dynamics in replicate anaerobic digesters exposed sequentially to increasing organic loading rate, acidosis, and process recovery. Biotechnol Biofuels.

[38] Poirier S, Desmond-Le Quéméner E, Madigou C, Bouchez T, Chapleur O (2016). Anaerobic digestion of biowaste under extreme ammonia concentration: identification of key microbial phylotypes. Bioresour Technol.

[39] Christou ML, Vasileiadis S, Karpouzas DG, Angelidaki I, Kotsopoulos TA (2021). Effects of organic loading rate and hydraulic retention time on bioaugmentation performance to tackle ammonia inhibition in anaerobic digestion. Bioresour Technol.

[40] Romsaiyud A, Songkasiri W, Nopharatana A, Chaiprasaert P (2009). Combination effect of pH and acetate on enzymatic cellulose hydrolysis. J Environ Sci.

[41] Fu X, Liu Z, Zhu C, Mou H, Kong Q (2019). Nondigestible carbohydrates, butyrate, and butyrate-producing bacteria. Crit Rev Food Sci Nutr.

[42] Lang K, Schuldes J, Klingl A, Poehlein A, Daniel R, Brune A (2015). New mode of energy metabolism in the seventh order of methanogens as revealed by comparative genome analysis of “Candidatus Methanoplasma termitum”. Appl Environ Microbiol.

[43] Tian H, Treu L, Konstantopoulos K, Fotidis IA, Angelidaki I (2019). 16s rRNA gene sequencing and radioisotopic analysis reveal the composition of ammonia acclimatized methanogenic consortia. Bioresour Technol.

[44] Lv Z, Leite AF, Harms H, Richnow HH, Liebetrau J, Nikolausz M (2014). Influences of the substrate feeding regime on methanogenic activity in biogas reactors approached by molecular and stable isotope methods. Anaerobe.

[45] Zielińska M, Cydzik-Kwiatkowska A, Zieliński M, Dębowski M (2013). Impact of temperature, microwave radiation and organic loading rate on methanogenic community and biogas production during fermentation of dairy wastewater. Bioresour Technol.

[46] Lebiocka M, Montusiewicz A, Cydzik-Kwiatkowska A (2018). Effect of bioaugmentation on biogas yields and kinetics in anaerobic digestion of sewage sludge. Int J Environ Res Public Health.

[47] Nie E, He P, Zhang H, Hao L, Shao L, Lü F (2021). How does temperature regulate anaerobic digestion?. Renew Sustain Energy Rev.

[48] Calusinska M, Goux X, Fossépré M, Muller EEL, Wilmes P, Delfosse P (2018). A year of monitoring 20 mesophilic full-scale bioreactors reveals the existence of stable but different core microbiomes in bio-waste and wastewater anaerobic digestion systems. Biotechnol Biofuels.

[49] Ma G, Chen Y, Ndegwa P (2021). Association between methane yield and microbiota abundance in the anaerobic digestion process: a meta-regression. Renew Sustain Energy Rev.

[50] CHEMANALYST. Real-time price movement of 200+ chemical and petrochemical products for informed purchase decisions. Mark Overv. 2022;38.

[51] Ni K, Köster JR, Seidel A, Pacholski A (2015). Field measurement of ammonia emissions after nitrogen fertilization-A comparison between micrometeorological and chamber methods. Eur J Agron.

[52] Rahman N, Forrestal PJ (2021). Ammonium fertilizer reduces nitrous oxide emission compared to nitrate fertilizer while yielding equally in a temperate grassland. Agriculture.

[53] Kim JM, To TK, Matsui A, Tanoi K, Kobayashi NI, Matsuda F (2017). Acetate-mediated novel survival strategy against drought in plants. Nat Plants.

[54] Utsumi Y, Utsumi C, Tanaka M, Van HC, Takahashi S, Matsui A (2019). Acetic acid treatment enhances drought avoidance in cassava (Manihot esculenta crantz). Front Plant Sci.

[55] Rahman MM, Mostofa MG, Rahman MA, Islam MR, Keya SS, Das AK (2019). Acetic acid: a cost-effective agent for mitigation of seawater-induced salt toxicity in mung bean. Sci Rep.

[56] Goux X, Calusinska M, Fossépré M, Benizri E, Delfosse P (2016). Start-up phase of an anaerobic full-scale farm reactor—appearance of mesophilic anaerobic conditions and establishment of the methanogenic microbial community. Bioresour Technol.

[57] Mayer F, Gerin PA, Noo A, Lemaigre S, Stilmant D, Schmit T (2014). Assessment of energy crops alternative to maize for biogas production in the Greater Region. Bioresour Technol.

[58] Van Kessel JS, Reeves JB (2000). On-farm quick tests for estimating nitrogen in dairy manure. J Dairy Sci.

[59] Hecht M, Clemens P, Wulf S. Entwicklung eines einfachen und für den Landwirt durchführbaren Verfahrens zur Überwachung der Prozessstabilität in landwirtschaftlichen Biogasanlagen. Schriftenr des Lehr- und Forschungsschwerpunktes USL. 2007;Nr. 151:51.

[60] Adam G, Lemaigre S, Goux X, Delfosse P, Romain AC (2015). Upscaling of an electronic nose for completely stirred tank reactor stability monitoring from pilot-scale to real-scale agricultural co-digestion biogas plant. Bioresour Technol.

[61] Edgar RC (2010). Search and clustering orders of magnitude faster than BLAST. Bioinformatics.

[62] Singh A, Schnürer A (2022). AcetoBase Version 2: a database update and re-analysis of formyltetrahydrofolate synthetase amplicon sequencing data from anaerobic digesters. Database.

[63] Westerholm M, Calusinska M, Dolfing J (2022). Syntrophic propionate-oxidizing bacteria in methanogenic systems. FEMS Microbiol Rev.

[64] Schloss PD, Westcott SL, Ryabin T, Justine RH, Hartmann M, Hollister EB (2009). Introducing mothur: open-source, platform-independent, community-supported software for describing and comparing microbial communities. Appl Environ Microbiol.

[65] Dixon P (2003). VEGAN, a package of R functions for community ecology. J Veg Sci.

