# Supplementary material for: Potential of acetic acid to restore methane production in anaerobic reactors critically intoxicated by ammonia as evidenced by metabolic and microbial monitoring
Source: Biotechnol Biofuels Bioprod. 2023 Dec 2;16:188. doi: 10.1186/s13068-023-02438-5 (PMC10693713; doi:10.1186/s13068-023-02438-5)
Supplement: Supplementary file 1 — Additional file 1: Table S1. Taxonomic affiliation of the top 50 bacterial OTUs presented in Fig. 4. Table S2. Composition of the dried sugar beet pulp pellets used to feed the anaerobic reactors. Fig. S1. Canonical correspondence analysis (CCA) ordination diplot for the top 50 more abundant bacterial OTUs. Data from all the reactors studied (the LNR and the three HNRs, 12 timepoints for each of them) were used for this analysis. Light-grey vectors represent process parameters influencing the microbial community, such as the pH (pH), volatile solids (VS), total solids (TS), total ammonia nitrogen (TAN), free ammonia nitrogen (FAN), acetic acid concentration (Acet. Ac.) and total inorganic carbon (TIC). Fig. S2. Canonical correspondence analysis (CCA) ordination diplot for the archaeal genera. Data from all the reactors studied (the LNR and the three HNRs, 12 timepoints for each of them) were used for this analysis. Light grey vectors represent process parameters influencing the microbial community, such as the pH (pH), total solids (TS), total ammonia nitrogen (TAN), free ammonia nitrogen (FAN), total inorganic carbon (TIC), recirculated low nitrogen reactor effluents (LNR Effluent), sugar beet pulp organic loading rate (Pulp. Input), acetic acid concentration (Acet. Ac.) and produced biogas volume (biogas). [file 13068_2023_2438_MOESM1_ESM.docx]

**Additional file 1**

**Potential of acetic acid to restore methane production in anaerobic reactors critically intoxicated by ammonia, as evidenced by metabolic and microbial monitoring**

Sébastien Lemaigre^a*^, Patrick A. Gerin^b^, Gilles Adam^a^, Dominika Klimek ^a^, Xavier Goux ^a^, Malte Herold^a^, Zuzana Frkova^a^, Magdalena Calusinska ^a^, Philippe Delfosse^c^

^a^ Luxembourg Institute of Science and Technology, Environmental Research and Innovation Department, Rue du Brill 41, L-4422 Belvaux, Luxembourg

^b^ Université catholique de Louvain, Earth & Life Institute, Bioengineering, Croix du Sud 2, box L7.05.19, B-1348 Louvain-la-Neuve, Belgium

^c^ Université du Luxembourg, Campus Belval, Maison du Savoir, Avenue de l’Université 2, L-4365 Esch-sur-Alzette

^*^ Corresponding author: Sebastien Lemaigre, [sebastien.lemaigre@list.lu](mailto:sebastien.lemaigre@list.lu), Luxembourg Institute of Science and Technology, Environmental Research and Innovation Department, Rue du Brill 41, L-4422 Belvaux, Luxembourg

**Table S1.** Taxonomic affiliation of the top 50 bacterial OTUs presented in Figure 4.

|  |  | |  | |  | |  | |  | |
| --- | --- | --- | --- | --- | --- | --- | --- | --- | --- | --- |
| OTUs | Phylum | | Class | | Order | | Family | | Genus | |
| OTU_1 | *Bacteroidota* (100) | | *Bacteroidia* (100) | | *Bacteroidales* (100) | | *Paludibacteraceae* (100) | | uncultured (100) | |
| OTU_10 | *Bacteroidota* (100) | | *Bacteroidia* (100) | | *Bacteroidales* (100) | | *Bacteroidaceae* (100) | | *Bacteroides* (100) | |
| OTU_100 | *Firmicutes* (100) | | *Clostridia* (100) | | *Oscillospirales* (99) | | *Oscillospirales*_unclassified (99) | | *Oscillospirales*_unclassified (99) | |
| OTU_11 | *Firmicutes* (100) | | *Clostridia* (100) | | *Lachnospirales* (100) | | *Lachnospiraceae* (100) | | *Lachnospiraceae*_unclassified (100) | |
| OTU_113 | *Firmicutes* (100) | | *Clostridia* (100) | | *Clostridia*_or (99) | | *Hungateiclostridiaceae* (99) | | *Hungateiclostridiaceae*_unclassified (99) | |
| OTU_12 | *Firmicutes* (100) | | *Limnochordia* (100) | | *MBA03* (100) | | *MBA03*_fa (100) | | *MBA03*_ge (100) | |
| OTU_13 | *Firmicutes* (100) | | *Clostridia* (99) | | *Caldicoprobacterales* (80) | | *Caldico probacteraceae* (80) | | *Caldicoprobacter* (80) | |
| OTU_14 | *Desulfobacterota* (100) | | *Syntrophia* (100) | | *Syntrophales* (100) | | *Smithellaceae* (100) | | *Smithella* (100) | |
| OTU_15 | *Firmicutes* (100) | | *Clostridia* (100) | | *Caldicoprobacterales* (100) | | *Caldicoprobacteraceae* (100) | | *Caldicoprobacter* (100) | |
| OTU_1524 | *Cloacimonadota* (100) | | *Cloacimonadia* (100) | | *Cloacimonadales* (100) | | *Cloacimonadaceae* (100) | | *Candidatus*_Cloacimonas (100) | |
| OTU_16 | *Firmicutes* (100) | | *Clostridia* (100) | | *Caldicoprobacterales* (100) | | *Caldicoprobacteraceae* (100) | | *Caldicoprobacter* (100) | |
| OTU_17 | *Bacteroidota* (100) | | *Bacteroidia* (100) | | *Bacteroidales* (100) | | *Rikenellaceae* (100) | | *DMER64* (100) | |
| OTU_1797 | *Bacteroidota* (100) | | *Bacteroidia* (100) | | *Bacteroidales* (100) | | *Prolixibacteraceae* (100) | | uncultured (99) | |
| OTU_18 | *Firmicutes* (100) | | *Limnochordia* (100) | | *Limnochordales* (100) | | uncultured (100) | | uncultured (100) | |
| OTU_19 | *Bacteroidota* (100) | | *Bacteroidia* (100) | | *Bacteroidales* (100) | | *Paludibacteraceae* (100) | | *Paludibacter* (100) | |
| OTU_2 | *Bacteroidota* (100) | | *Bacteroidia* (100) | | *Bacteroidales* (100) | | *Prolixibacteraceae* (100) | | uncultured (100) | |
| OTU_20 | *Spirochaetota* (99) | | *Spirochaetia* (99) | | *Spirochaetales* (99) | | *Spirochaetaceae* (99) | | *Sphaerochaeta* (97) | |
| OTU_21 | *Bacteroidota* (100) | | *Bacteroidia* (100) | | *Bacteroidales* (100) | | *Bacteroidetes_vadinHA17* (100) | | *Bacteroidetes_vadinHA17*_ge (100) | |
| OTU_2391 | *Firmicutes* (96) | | *Limnochordia* (82) | | *Limnochordia*_unclassified (82) | | *Limnochordia*_unclassified (82) | | *Limnochordia*_unclassified (82) | |
| OTU_24 | *Bacteroidota* (100) | | *Bacteroidia* (100) | | *Bacteroidales* (100) | | *Prevotellaceae* (100) | | *Prevotella* (99) | |
| OTU_25 | *Planctomycetota* (100) | | *Phycisphaerae* (100) | | *MSBL9* (100) | | *SG8-4* (100) | | *SG8-4*_ge (100) | |
| OTU_27 | *Cloacimonadota* (100) | | *Cloacimonadia* (100) | | *Cloacimonadales* (100) | | *Cloacimonadaceae* (100) | | *W5* (100) | |
| OTU_28 | *Cloacimonadota* (100) | | *Cloacimonadia* (100) | | *Cloacimonadales* (100) | | *Cloacimonadaceae* (100) | | *Candidatus*_Cloacimonas (98) | |
| OTU_29 | *Acidobacteriota* (100) | | *Aminicenantia* (99) | | *Aminicenantales* (99) | | *Aminicenantales*_fa (99) | | *Aminicenantales*_ge (99) | |
| OTU_3 | *Spirochaetota* (100) | | *Spirochaetia* (100) | | *Spirochaetales* (100) | | *Spirochaetaceae* (100) | | uncultured (100) | |
| OTU_30 | *Chloroflexi* (100) | | *Anaerolineae* (100) | | *SJA-15* (100) | | *SJA-15*_fa (100) | | *SJA-15*_ge (100) | |
| OTU_31 | *Firmicutes* (100) | | *Limnochordia* (100) | | *Limnochordales* (100) | | uncultured (100) | | uncultured (100) | |
| OTU_32 | *Acidobacteriota* (100) | | *c5LKS83* (100) | | *c5LKS83*_or (100) | | *c5LKS83*_fa (100) | | *c5LKS83*_ge (100) | |
| OTU_33 | *Firmicutes* (100) | | *Clostridia* (100) | | *Clostridia*_or (100) | | *Hungateiclostridiaceae* (100) | | *HN-HF0106* (100) | |
| OTU_35 | *Firmicutes* (100) | | *Clostridia* (97) | | *Lachnospirales* (97) | | *Lachnospiraceae* (97) | | *Lachnospiraceae*_unclassified (97) | |
| OTU_36 | *Spirochaetota* (98) | | *Spirochaetia* (98) | | *Spirochaetales* (98) | | *Spirochaetaceae* (98) | | *Sphaerochaeta* (93) | |
| OTU_37 | *Bacteroidota* (100) | | *Bacteroidia* (100) | | *Bacteroidales* (100) | | *Dysgonomonadaceae* (100) | | *Proteiniphilum* (100) | |
| OTU_4 | *Firmicutes* (100) | | *Clostridia* (99) | | *Lachnospirales* (99) | | *Lachnospiraceae* (99) | | *Lachnospiraceae*_unclassified (99) | |
| OTU_40 | *Actinobacteriota* (100) | | *Actinobacteria* (100) | | *Micrococcales* (98) | | *Bogoriellaceae* (88) | | *Georgenia* (88) | |
| OTU_43 | *Firmicutes* (100) | | *Clostridia* (100) | | *Lachnospirales* (100) | | *Lachnospiraceae* (100) | | *Lachnospiraceae*_unclassified (100) | |
| OTU_44 | *Patescibacteria* (100) | | *ABY1* (100) | | *Candidatus*_Falkowbacteria (100) | | *Candidatus*_Falkowbacteria*_*fa (100) | | *Candidatus*_Falkowbacteria_ge (100) | |
| OTU_45 | *Bacteroidota* (100) | | *Bacteroidia* (100) | | *Bacteroidales* (100) | | *Dysgonomonadaceae* (100) | | *Dysgonomonadaceae*_unclassified (100) | |
| OTU_46 | *Firmicutes* (100) | | *Limnochordia* (100) | | uncultured (100) | | uncultured_fa (100) | | uncultured_ge (100) | |
| OTU_47 | *Firmicutes* (100) | | *Clostridia* (100) | | *Peptostreptococcales-Tissierellales* (100) | | *Peptostreptococcales-Tissierellales*_fa (100) | | *Peptostreptococcales-Tissierellales*_fa_unclassified (100) | |
| OTU_5 | *Bacteroidota* (100) | | *Bacteroidia* (100) | | *Bacteroidales* (100) | | *Dysgonomonadaceae* (100) | | *Proteiniphilum* (100) | |
| OTU_52 | *Firmicutes* (100) | | *Clostridia* (97) | | *Lachnospirales* (97) | | *Defluviitaleaceae* (88) | | *Defluviitalea* (88) | |
| OTU_53 | *Firmicutes* (100) | | *Clostridia* (100) | | *Peptostreptococcales-Tissierellales* (100) | | *Sedimentibacteraceae* (100) | | *Sedimentibacter* (100) | |
| OTU_576 | *Firmicutes* (99) | | *Clostridia* (99) | | *Lachnospirales* (99) | | *Lachnospiraceae* (99) | | *Lachnospiraceae*_unclassified (99) | |
| OTU_58 | *Firmicutes* (100) | | *Clostridia* (100) | | *Clostridia*_or (97) | | *Hungateiclostridiaceae* (97) | | *Hungateiclostridiaceae*_unclassified (97) | |
| OTU_6 | *Firmicutes* (100) | | *Limnochordia* (92) | | *M55-D21* (84) | | *M55-D21*_fa (84) | | *M55-D21*_ge (84) | |
| OTU_7 | *Bacteroidota* (100) | | *Bacteroidia* (100) | | *Bacteroidales* (100) | | *Dysgonomonadaceae* (100) | | *Fermentimonas* (100) | |
| OTU_760 | *Firmicutes* (100) | | *Limnochordia* (100) | | *MBA03* (98) | | *MBA03*_fa (98) | | *MBA03*_ge (98) | |
| OTU_8 | *Chloroflexi* (100) | | *Anaerolineae* (100) | | *Anaerolineales* (100) | | *Anaerolineaceae* (100) | | uncultured (100) | |
| OTU_83 | *Firmicutes* (100) | | *Clostridia* (100) | | *Peptostreptococcales-Tissierellales* (100) | | *Peptostreptococcales-Tissierellales*_fa (100) | | *Tepidimicrobium* (99) | |
| OTU_97 | *Firmicutes* (100) | | *Clostridia* (99) | | *Caldicoprobacterales* (95) | | *Caldicoprobacteraceae* (95) | | *Caldicoprobacter* (95) | |
|  |  |  | |  | |  | |  | |  |

The numbers in brackets indicate the percentages of sequence identity.

**Table S2.** Composition of the dried sugar beet pulp pellets used to feed the anaerobic reactors.

|  | **Dry basis (%)** | **As fed (%)** |
| --- | --- | --- |
| Total solids |  | 86.86 |
| Moisture |  | 13.14 |
| Volatile solids | 92.06 | 79.96 |
| Ash | 7.94 | 6.90 |
| Crude protein | 8.29 | 7.20 |
| Crude fibre | 16.97 | 14.74 |
| Acid detergent fibre (ADF) | 26.68 | 23.17 |
| Total digestible nutrients (TDN) | 69.40 | 60.28 |
| Fat | 1.09 | 0.95 |
| Nitrogen-free extract (NFE) | 66.09 | 57.41 |
| Calcium | 1.00 | 0.87 |
| Phosphorus | 0.07 | 0.06 |
| Potassium | 0.56 | 0.49 |
| Reducing sugars | 2.60 | 2.26 |
| Sucrose | 8.96 | 7.78 |
| Total sugars as invert (TSI) | 8.23 | 7.15 |

**
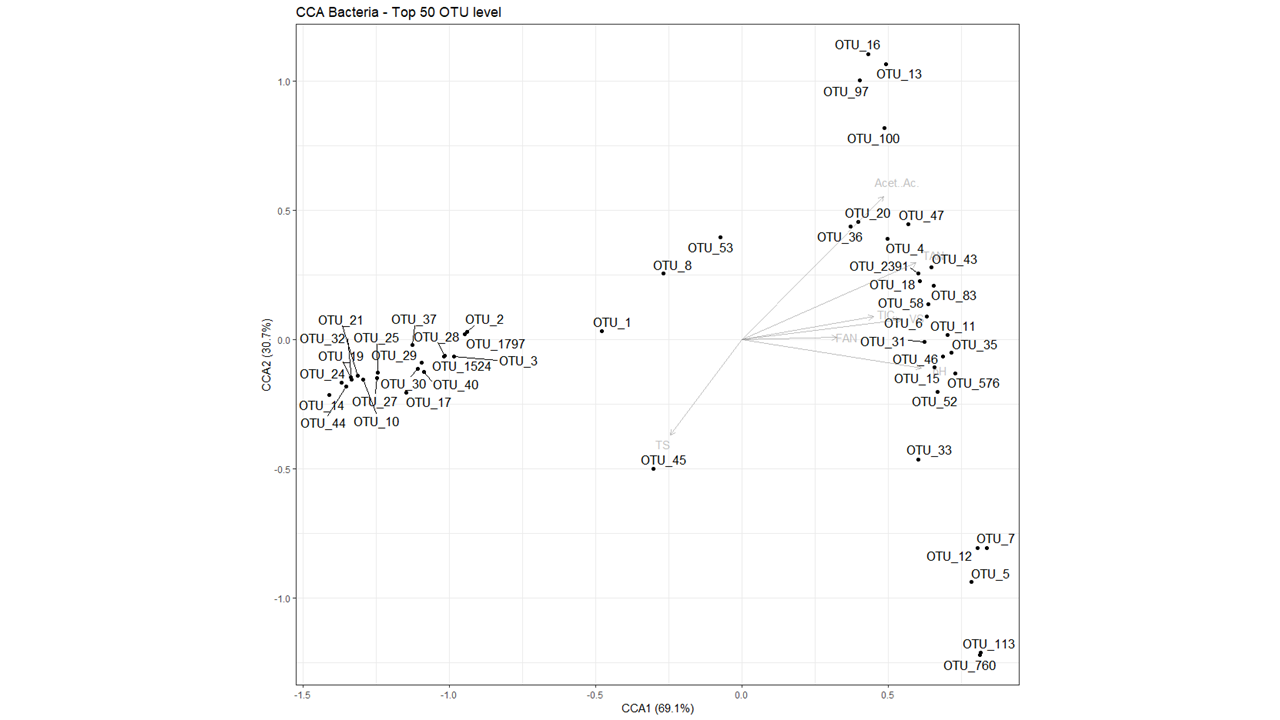
**

**Fig. S1.** Canonical correspondence analysis (CCA) ordination diplot for the top 50 more abundant bacterial OTUs. Data from all the reactors studied (the LNR and the three HNRs, 12 time points for each of them) were used for this analysis. Light-grey vectors represent process parameters influencing the microbial community such as the pH (pH), volatile solids (VS), total solids (TS), total ammonia nitrogen (TAN), free ammonia nitrogen (FAN), acetic acid concentration (Acet. Ac.) and total inorganic carbon (TIC).

**
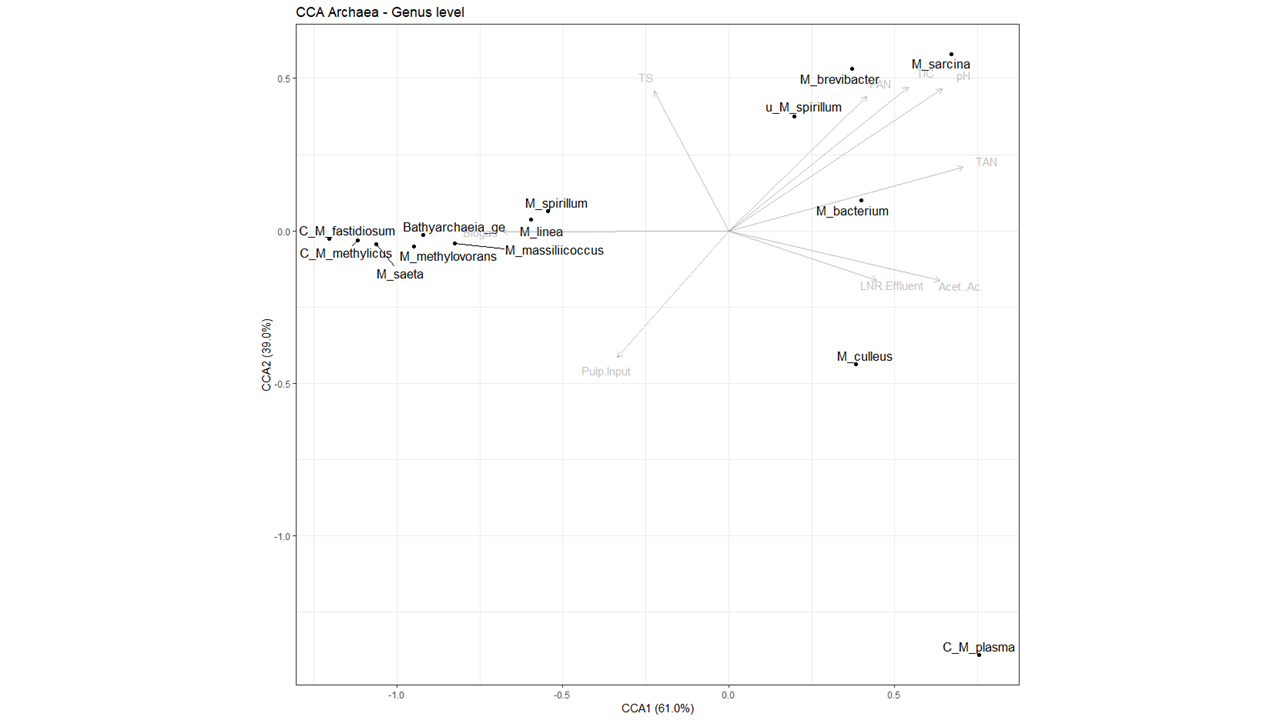
**

**Fig. S2.** Canonical correspondence analysis (CCA) ordination diplot for the archaeal genera. Data from all the reactors studied (the LNR and the three HNRs, 12 time points for each of them) were used for this analysis. Light grey vectors represent process parameters influencing the microbial community such as the pH (pH), total solids (TS), total ammonia nitrogen (TAN), free ammonia nitrogen (FAN), total inorganic carbon (TIC), recirculated low nitrogen reactor effluents (LNR Effluent), sugar beet pulp organic loading rate (Pulp. Input), acetic acid concentration (Acet. Ac.) and produced biogas volume (biogas).
